# The biological significance of cuproptosis-key gene MTF1 in pan-cancer and its inhibitory effects on ROS-mediated cell death of liver hepatocellular carcinoma

**DOI:** 10.1007/s12672-023-00738-8

**Published:** 2023-06-28

**Authors:** Liying Song, Rong Zeng, Keda Yang, Wei Liu, Zhijie Xu, Fanhua Kang

**Affiliations:** 1 Department of Pharmacy, The Third Xiangya Hospital, Central South University, Changsha, Hunan China; 2 General Surgery Department, Second Xiangya Hospital, Central South University, Changsha, Hunan China; 3 Department of Pathology, Xiangya Hospital, Central South University, Changsha, Hunan China; 4 National Clinical Research Center for Geriatric Disorders, Xiangya Hospital, Central South University, Changsha, Hunan China; 5 Department of Orthopedic Surgery, The Second Hospital University of South China, Hengyang, Hunan China; 6 Department of Pathology, Xiangya Changde Hospital, Changde, Hunan China

**Keywords:** MTF1, Cell death, Metabolism, Immunity, Cancer

## Abstract

Metal regulatory transcription factor 1 (MTF1) has been reported to be correlated with several human diseases, especially like cancers. Exploring the underlying mechanisms and biological functions of MTF1 could provide novel strategies for clinical diagnosis and therapy of cancers. In this study, we conducted the comprehensive analysis to evaluate the profiles of MTF1 in pan-cancer. For example, TIMER2.0, TNMplot and GEPIA2.0 were employed to analyze the expression values of MTF1 in pan-cancer. The methylation levels of MTF1 were evaluated via UALCAN and DiseaseMeth version 2.0 databases. The mutation profiles of MTF1 in pan-cancers were analyzed using cBioPortal. GEPIA2.0, Kaplan–Meier plotter and cBioPortal were also used to explore the roles of MTF1 in cancer prognosis. We found that high MTF1 expression was related to poor prognosis of liver hepatocellular carcinoma (LIHC) and brain lower grade glioma (LGG). Also, high expression level of MTF1 was associated with good prognosis of kidney renal clear cell carcinoma (KIRC), lung cancer, ovarian cancer and breast cancer. We investigated the genetic alteration and methylation levels of MTF1 between the primary tumor and normal tissues. The relationship between MTF1 expression and several immune cells was analyzed, including T cell CD8 + and dendritic cells (DC). Mechanically, MTF1-interacted molecules might participate in the regulation of metabolism-related pathways, such as peptidyl-serine phosphorylation, negative regulation of cellular amide metabolic process and peptidyl-threonine phosphorylation. Single cell sequencing indicated that MTF1 was associated with angiogenesis, DNA repair and cell invasion. In addition, in vitro experiment indicated knockdown of MTF1 resulted in the suppressed cell proliferation, increased reactive oxygen species (ROS) and promoted cell death in LIHC cells HepG2 and Huh7. Taken together, this pan-cancer analysis of MTF1 has implicated that MTF1 could play an essential role in the progression of various human cancers.

**Supplementary Information:**

The online version contains supplementary material available at 10.1007/s12672-023-00738-8.

## Introduction

The progression of cancer has been found to be correlated with the imbalance of gene regulation programs. Searching for new candidate genes that contribute to the cancer development would be meaningful for the early screening and understanding of tumors correlated regulatory pathways [1–3].

Metal regulatory transcription factor 1 (MTF1) has been found to be a zinc finger-containing transcription factor that regulates subcellular metal metabolism, such as copper, iron or zinc [4]. Structurally, MTF1 consists of a α/β N-terminal domain and a tetra-α helical C-terminal domain. Of note, N-terminal domains have the function of interacting with various rRNA methyltransferases [5]. MTF1 plays a crucial role in maintaining intracellular metal homeostasis and preventing cells from excessive metal damage [6]. Furthermore, MTF1 could be translocated into the nucleus, leading to the activation of its downstream genes, such as matrix metalloproteinases (MMPs), metal binding protein metallothionein (MT1) and so on [7]. Several seminal studies have delineated the unique functions of MTF1 in the development of various diseases, especially like cancers [8]. Acetylated METTL3 could enhance MTF1 mRNA stability by binding its mRNA and reducing the m6A modification, consequently facilitating cell proliferation in liver hepatocellular carcinoma (LIHC) [9]. He et al. showed that downregulated MTF1 could serve as an independent prognostic factor for gastric cancer patients [10]. Although the aberrantly expressed MTF1 has been detected in several cancers, its potential biological functions and underlying mechanisms have not been well investigated.

Here, we employed the TCGA dataset and some other bioinformatics tools to explore the regulation roles of MTF1 in a variety of cancers (Supplementary Table S1). We not only explored the expression levels of MTF1 in cancers, but also investigated the survival values, genetic alteration, methylation and enriched signaling pathways. These explorations have elucidated that MTF1 could play a vital part in the progression of various cancers.

## Materials and methods

### Gene expression analysis

Three databases, TIMER2.0 [11] (http://timer.cistrome.org/), TNMplot [12] (https://tnmplot.com/analysis/) and Gene Expression Profiling Interactive Analysis, version 2 (GEPIA2.0) [13] (http://gepia2.cancer-pku.cn/), were applied to evaluate the MTF1 expression between the normal groups and the tumor groups. Apart from the TCGA samples, GEPIA2.0 database also collected the data from genotype-tissue expression dataset (GTEx). In GEPIA2.0, The p value was set as: p < 0.05 and the cutoff of |Log_2_FC| was 0.1. Meanwhile, GEPIA2.0 was used to evaluate the expression of MTF1 and pathological stages in TCGA pan-cancer. In addition, the UALCAN [14] (https://ualcan.path.uab.edu/) was employed to explore the methylation levels of MTF1 in TCGA datasets. In addition, The GEPIA2.0 and Kaplan–Meier plotter [15] (http://kmplot.com/analysis/) have confirmed the prognostic values of MTF1 expression in several cancers.

### Immunohistochemistry (IHC) staining

From Human Protein Atlas (HPA) [16] (https://www.proteinatlas.org/) database, antibody HPA028689 was applied to obtain the Immunohistochemistry (IHC) staining of MTF1 between normal tissues and tumor tissues, including 11 kidney cancer tissues, 12 testis cancer tissues and 12 colon cancer tissues. We used HPA to identify the expression profiles of MTF1 in several cancers, such as renal cancer, testicular germ cell tumors (TGCT) and colon adenocarcinoma (COAD).

### Genetic alteration analysis

The cBioPortal [17] (http://www.cbioportal.org/) was applied to obtain the mutation profiles of MTF1 in TCGA pan-cancer, including alteration frequency, mutation type and mutated site. The effect of MTF1 genetic alteration on survival data for cancer patients were also downloaded from cBioPortal. The survival analysis mainly included disease-free survival (DFS), disease-specific survival (DSS), overall survival (OS) and progression-free survival (PFS). The FAQs section in cBioPortal provided the detailed mutation annotation information, including identification, sites and regions, types, and clinical prognosis. For the prognosis analysis, STATUS represents patients’ survival status with “0” meaning “living” or “1” meaning “deceased”, and MONTHS represents the time from the start of diagnosis to the end of follow-up.

### Immune analysis

The TIMER2.0 database was employed to evaluate the correlation between MTF1 expression and multiple immune infiltrating cells across TCGA pan-cancer. T cell CD8 + cells, dendritic cells (DC), NK cell, T cell regulatory (Tregs), cancer-associated fibroblast (CAF), neutrophil, B cell and macrophage were searched for further evaluations.

### The analysis of single cell sequencing

The single cell sequencing could be used for the functional analysis of candidate genes in human diseases at a single cell level [18–20]. The correlation heatmap between MTF1 expression and functional status were obtained from CancerSEA [21] (http://biocc.hrbmu.edu.cn/CancerSEA/). The t-SNE pictures were downloaded from the CancerSEA tool. The CancerSEA website collected 41,900 cells from 25 cancer types. Meanwhile, GSVA and Spearman’s correlations were used to analyze the functional states and correlations between the biological activities and MTF1 expressions, respectively. The significant gene-state associations were identified with FDR < 0.05 and correlation > 0.3.

### MTF1-related gene enrichment evaluations

The STRING [22] (https://string-db.org/) website was applied for protein–protein network evaluation. The main settings were provided as follows: meaning of network edges (“evidence”), minimum required interaction score [“Medium confidence (0.400)”], max number of interactors to show (“no more than 10 interactors” in 1st shell and “no more than 20 interactors” in 2nd shell). And we employed the Top # similar Genes in GEPIA2.0 to download the top 100 MTF1-associated genes across TCGA pan-cancer and the corresponding normal tissues. Meantime, we performed Gene Ontology (GO) analysis to evaluate the possible pathways regulated by MTF1-associated molecules. After uploading the top 100 MTF1-associated molecules, the GO enrichment results were automatically generated by Xiantao Xueshu (https://www.xiantaozi.com/login). P < 0.05 was regarded as statistically significant.

### Cell culture

The human liver LIHC cells HepG2 and Huh7 were gift from Xiangya Cancer Center, Xiangya Hospital, Central South University [23]. These cells were cultured in the Dulbecco’s modified Eagle’s medium (DMEM) at 37 °C with 5% CO2. The 10% fetal bovine serum and 1% penicillin/streptomycin were supplemented in the DMEM medium.

### Cell transfection and western blot

Using lipofectamine 3000, the cells were transfected with MTF1 siRNAs (siMTF1-1: GGAAGATCCTCAACAGACA and siMTF1-2: GAAAGGTCATGATAACAAA). After then, as previously described [24, 25], the total protein expression levels were accessed using western blot.

In brief, cells were harvested at the indicated times and lysed in the lysis buffer (10 mM Tris–HCl, pH 8.0, 1 mM EDTA, 2% SDS, 5 mM DTT, 10 mM PMSF, proteinase inhibitor mix) for 30 min on ice. After centrifuged at 13,000 rpm for about 15 min, the total protein was quantified by BCA Assay Reagent (Pierce Chemical, Inc). Equal amounts of total proteins (50 μg) were resolved on a 10% gels of sodium dodecyl sulfate–polyacrylamide gel electrophoresis (SDS-PAGE) and transferred onto PVDF Transfer Membrane. After blocked with TBS buffer containing 5% skimed milk and 0.1% Tween 20, the PVDF membranes were incubated with with the indicated primary antibodies. At last, the bands were determined using the enhanced chemiluminescence detection kit (Pierce ECL, Thermo Scientific). The primary antibodies were displayed as follows: β-actin antibody (Santa Cruz, 8432) and MTF1 antibody (Proteintech, 25383-1-AP).

### CCK-8 cell proliferation assays

According to the protocols of cell counting kit-8 (CCK-8, Bimake, B34302), 2 × 10^4^ cells transfected with MTF1 siRNAs were plated into the 96-well plates, and cultured for the indicated times (1 day, 2 day, 3 day, 4 day and 5 day). Each groups contained five replicates. Then, 10 μL CCK-8 reagent was mixed into each well and incubated at 37 °C for about 2 h. The absorption peak intensity of reaction mixture was assessed using an absorbent photometer at 495 nm.

### Cell death assays

The cell death was analyzed by Trypan blue staining (Beyotime, C0011). According to the protocols of Trypan blue staining, 5 × 10^3^ cells were plated into the 24-well plates. Subsequent, the cells were enriched and re-suspended in 0.4% trypan blue reagent with serum-free medium for about 1 min. At last, a haemocytometer was used to count the death cells.

### Survival analysis

The GEPIA2.0 was used to download the survival map and the survival plots of cancer patients, including OS and DFS. The significance level was set as 0.05. The cutoff-high (50%) and cutoff-low (50%) values were employed for the expression thresholds between the high-expression cohorts and the low-expression cohorts. In TIMER2.0, The statistical significance for differentially expressed MTF1 was computed by Wilcoxon test. * p < 0.05; ** p < 0.01; *** p < 0.001. Student’s t-test, linear regression analysis, and Cox regression analysis were conducted when appropriate in other databases or in vitro expriments.

## Results

### The evaluation of MTF1 expression in pan-cancer

**Fig. 1:**
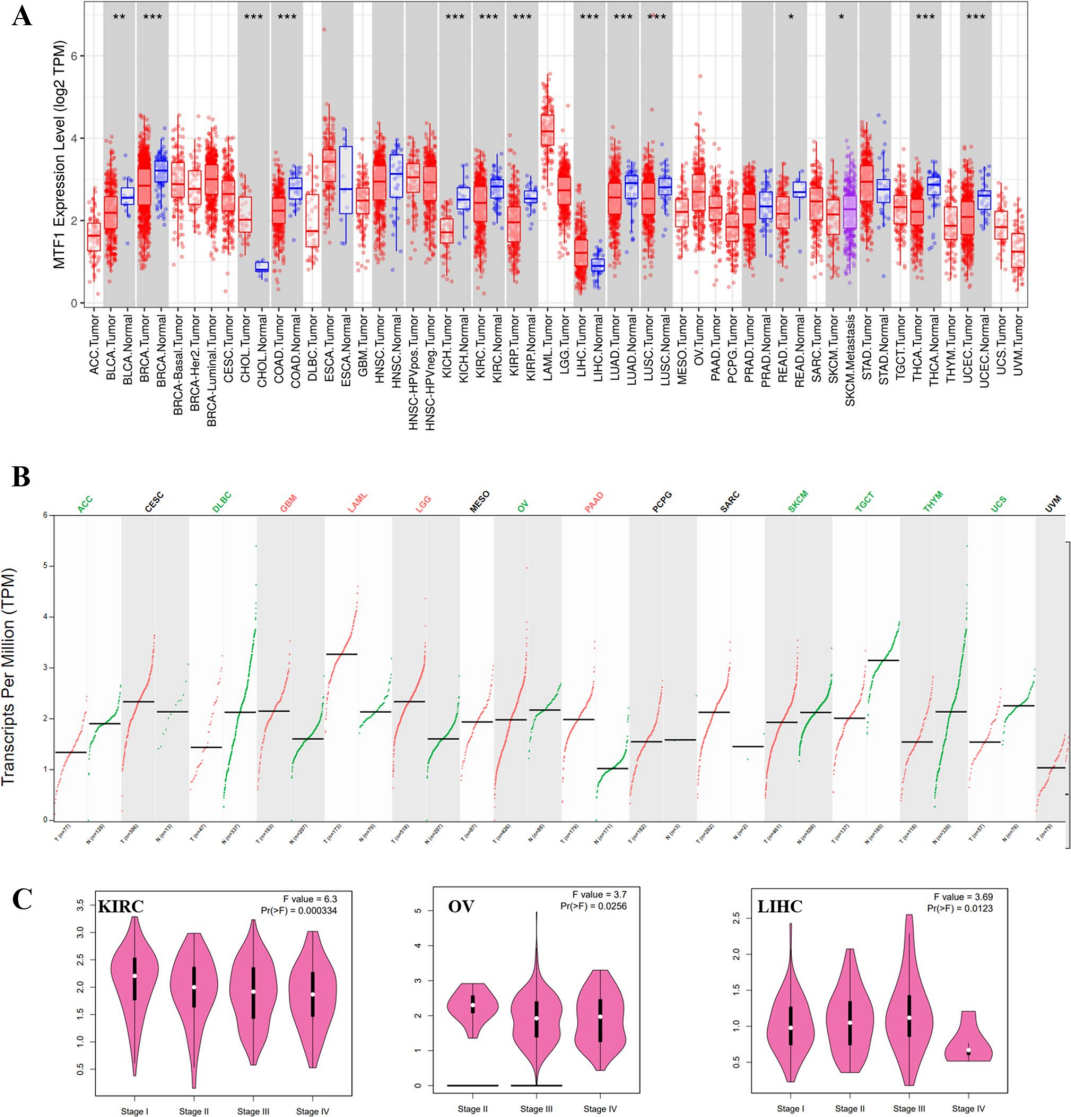
The expression profiles of MTF1 in human cancers. **A** TIMER2.0 displaying the MTF1 expression in TCGA tumors and the corresponding normal tissues. *p < 0.05; ** p < 0.01; ***p < 0.001. **B** GEPIA2.0 database showing the MTF1 expression in tumor tissues and the corresponding normal tissues. The red and green tumor names represented the over-expressed and low-expressed MTF1 in tumors, respectively. **C** The effect of MTF1 on the pathological stages in patients with KIRC, OV and LIHC

The TIMER2.0 database was used to analyze the MTF1 expression between tumor and normal tissues across TCGA datasets. The down-regulated expression levels of MTF1 could be identified in most of cancers, including breast invasive carcinoma (BRCA), COAD, kidney chromophobe (KICH), KIRC, kidney renal papillary cell carcinoma (KIRP), lung adenocarcinoma (LUAD), lung squamous cell carcinoma (LUSC), thyroid carcinoma (THCA), uterine corpus endometrial carcinoma (UCEC), bladder urothelial carcinoma (BLCA) and rectum adenocarcinoma (READ). In contrary, MTF1 displayed higher expression in cholangiocarcinoma (CHOL) and LIHC than the corresponding normal tissues (Fig. 1A). Given some normal tissues could not be obtained from the TIMER2.0 database, we further compared the MTF1 expression levels between tumor tissues and normal tissues by GEPIA2.0 that comprises the GTEx datasets. And we found that MTF1 expression was significantly up-regulated in glioblastoma multiforme (GBM), acute myeloid leukemia (LAML), brain lower grade glioma (LGG) and pancreatic adenocarcinoma (PAAD). At the same time, the expression levels of MTF1 in adrenocortical carcinoma (ACC), lymphoid neoplasm diffuse large B-cell lymphoma (DLBC), ovarian serous cystadenocarcinoma (OV), skin cutaneous melanoma (SKCM), TGCT, thymoma (THYM) and uterine carcinosarcoma (UCS) were lower than that in normal tissues (Fig. 1B). In addition, we used the GEO database to analyze the expression profiles of MTF1 in some cancers. Similarly, we confirmed the down-regulated MTF1 expression in adrenocortical carcinoma, colon cancer and kidney cancer (Supplementary Figure S1A-C). Moreover, we identified the up-regulated MTF1 expression in liver cancer and glioma (Supplementary Figure S1D-E). The GEPIA2.0 database was also applied to evaluate the links between MTF1 expression and the pathological stages. As shown in Fig. 1C, aberrant expression of MTF1 had obvious effects on the patients’ stages in KIRC, OV and LIHC. However, no obvious association could be identified between MTF1 levels and pathological stages in other cancers (Supplementary Figure S2A-T).

**Fig. 2:**
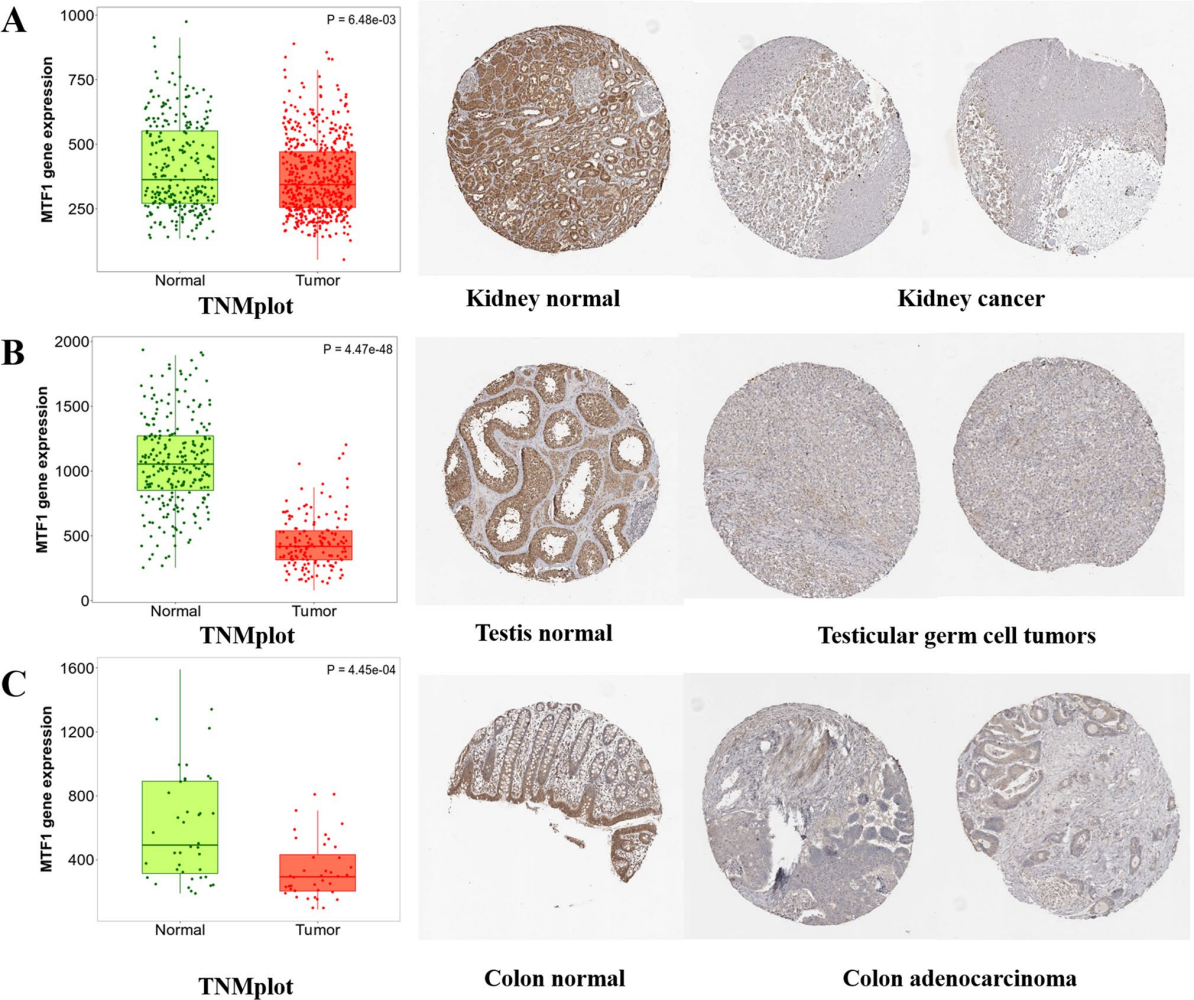
The different expression of MTF1 between normal and tumor tissues. **A**–**C** TNMplot and HPA databases showed the downregulated MTF1 in kidney cancer, testis cancer and colon cancer (p < 0.05)

In addition, we applied HPA database to further explore the protein levels of MTF1 in the cancers. MTF1 displayed strong or medium staining in normal kidney, testis and colon tissues, and weak or negative staining in the corresponding tumor tissues. The down-regulated protein levels of MTF1 in these cancers were consistent with the transcriptional levels of MTF1 obtained from the TNMplot (Fig. 2A–C). Meanwhile, the CPTAC from UALCAN database also showed MTF1 protein levels in multiple types of cancers. As the diagraph depicted, the MTF1 protein levels in UCEC, lung cancer and GBM were higher than that in normal groups. Whereas, the expression of MTF1 in head and neck cell carcinoma was lower than that in normal group (Supplementary Figure S3).

### The effect of MTF1 expression on the patients’ prognosis

**Fig. 3:**
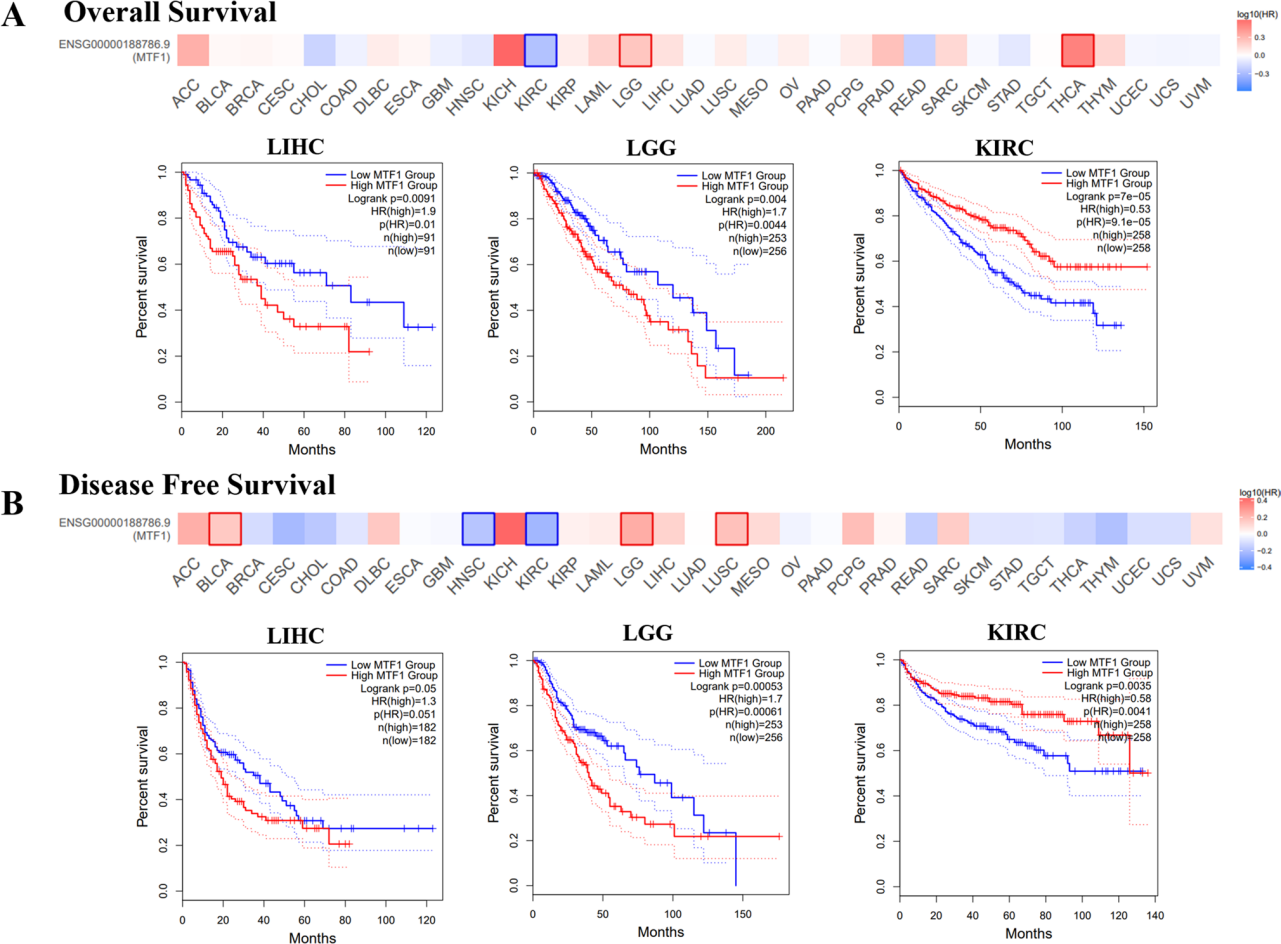
The prognostic values of MTF1 in pan-cancers. **A**–**B** The GEPIA2.0 database showed the effects of MTF1 expression on the overall survival (OS) and disease-free survival (DFS) across TCGA cancers

We divided the patients into highly-expressed MTF1 group and lowly-expressed MTF1 group to explore the effect of MTF1 expression levels on the OS and DFS. According to the results from GEPIA2.0 database, we found that high expression levels of MTF1 were related to the poor OS in LIHC (p = 0.0091) and LGG (p = 0.004), while high expression levels of MTF1 were linked to the good OS in KIRC (p = 7e-05) (Fig. 3A). Additionally, Fig. 3B has demonstrated that high expression levels of MTF1 were related to the poor DFS in LIHC (p = 0.05) and LGG (p = 0.00053) while high expression levels of MTF1 were linked to the good DFS in KIRC (p = 0.0035). By means of Kaplan–Meier plotter, we found that lung cancer patients with high expression levels of MTF1 displayed good OS, first progression (FP) and post-progression survival (PPS) (Supplementary Figure S4A). Also, the ovarian cancer patients with higher MTF1 expression displayed good OS, PPS and PFS (Supplementary Figure S4B). The breast cancer patients with high expression levels of MTF1 displayed good OS, PPS, relapse-free survival (RFS) and distant metastasis free survival (DMFS) (Supplementary Figure S4C). The liver cancer patients with high expression levels showed poor OS (Supplementary Figure S4D). Meanwhile, we explored the roles of MTF1 on the posttreatment prognosis of cancer patients. Kaplan–Meier analysis demonstrated that high MTF1 expression indicated favorable OS for the lung cancer patients treated with chemotherapy (Supplementary Figure S5A). High expression of MTF1 also showed good OS for the ovarian cancer patients treated with multiple chemotherapy agents, including gemcitabine, paclitaxel, platin and topotecan (Supplementary Figure S5B). Moreover, high MTF1 expression of breast cancer patients treated with chemotherapy or endocrine therapy displayed good OS (Supplementary Figure S5C). These results collectively demonstrated the important roles of aberrantly-expressed MTF1 on the prognosis of patients with cancers.

### The genetic alteration analysis of MTF1

**Fig. 4:**
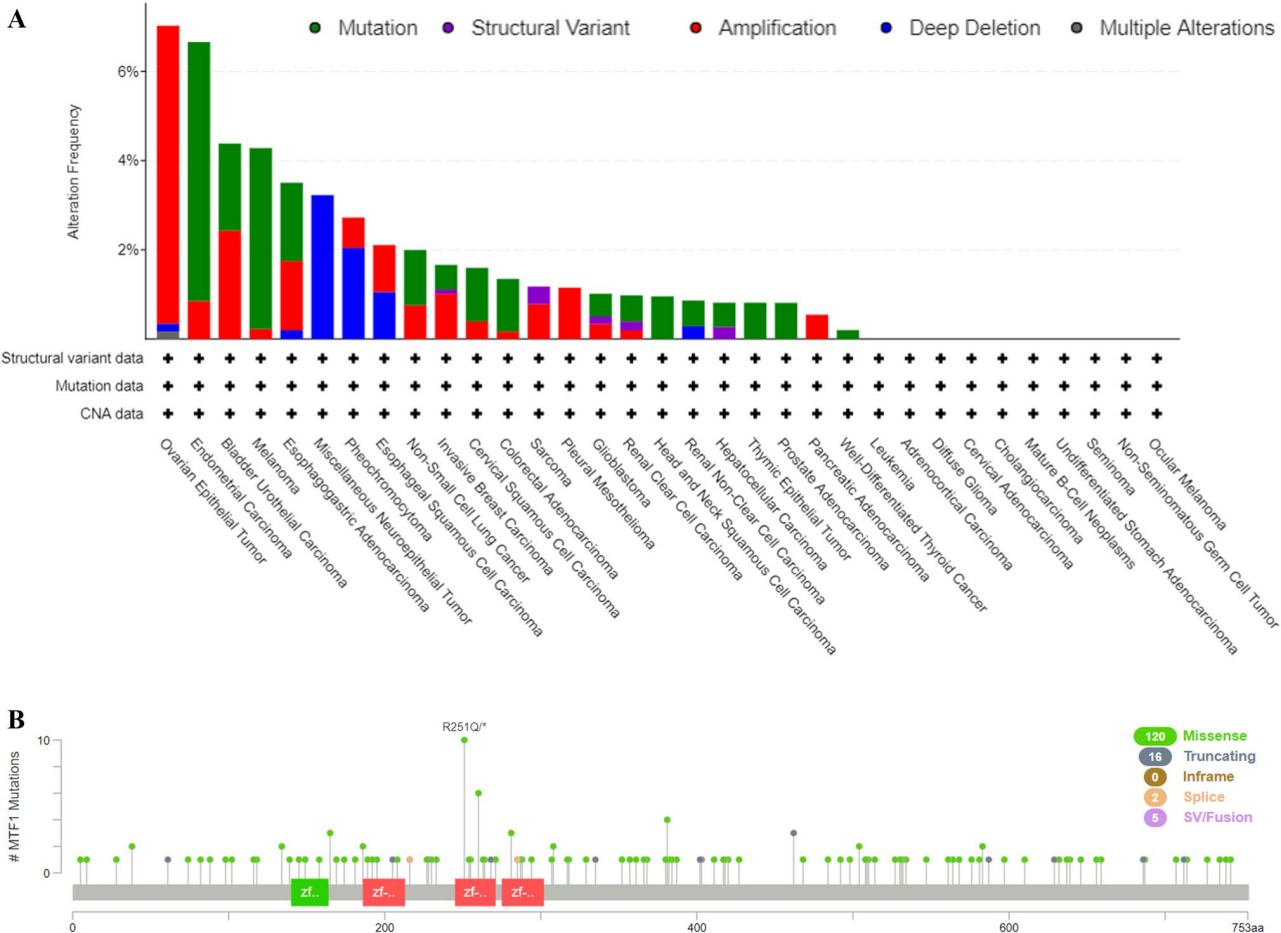
The cBioPortal indicated the mutation status of MTF1 in TCGA pan-cancer. **A**–**B** The diagrams displayed the alteration frequency of different mutation types and the distribution of mutation site on MTF1 sequence

**Fig. 5:**
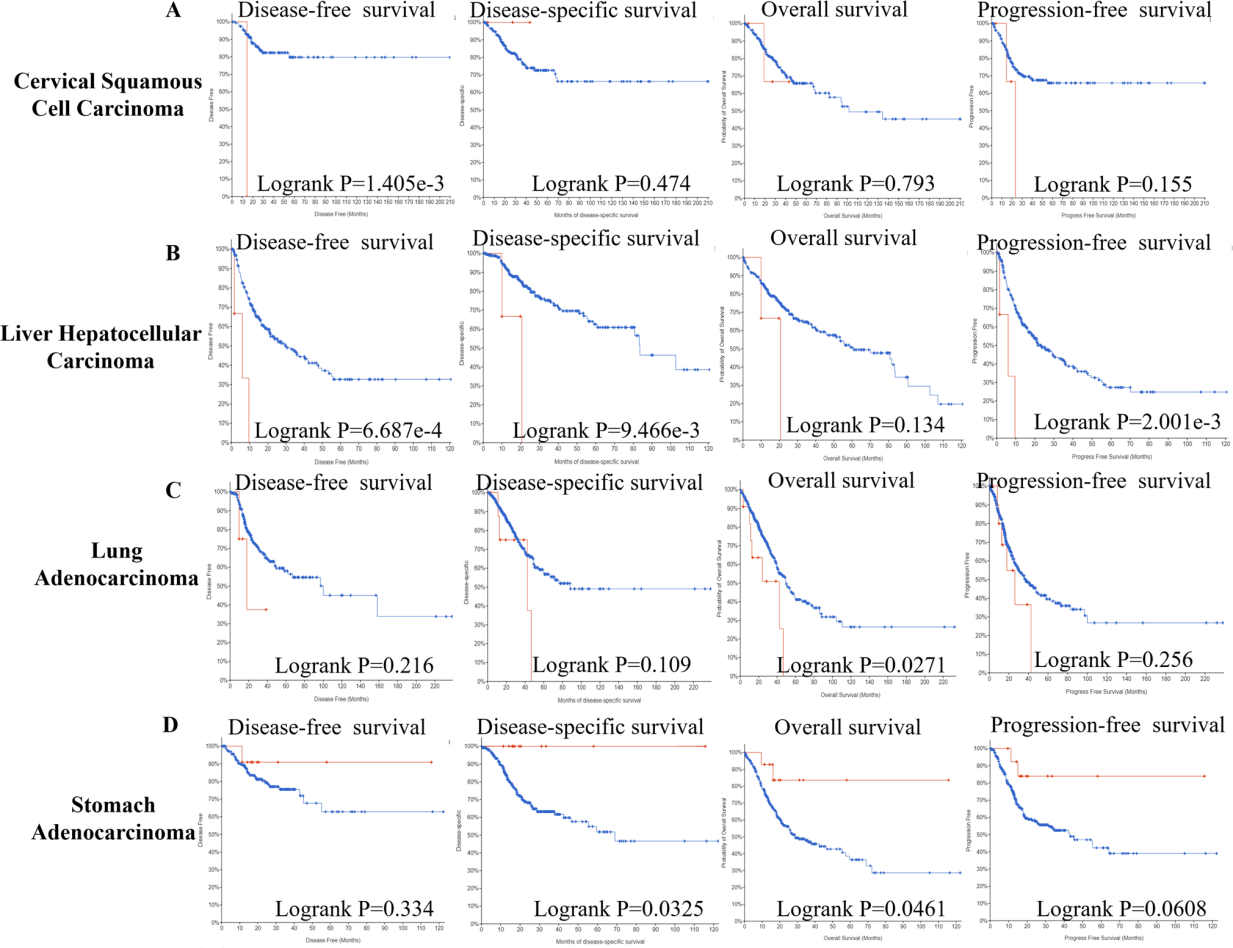
The cBioPortal indicated the prognostic values of MTF1 alteration in cancers. **A**–**D** These data showed the correlation between MTF1 mutation status and survival values (DSS, DFS, PFS and OS) in some cancers

The MTF1 genetic alteration analysis was explored by means of cBioPortal tool. And the histogram showed that the amplification frequency of MTF1 was the highest in ovarian epithelial tumor. And the mutation frequency of MTF1 was the highest in endometrial carcinoma (Fig. 4A). Additionally, we explored the mutation types and their location within MTF1 sequence. We found the types of MTF1 mutation mainly contained missense mutation, truncating mutation, splice mutation and fusion mutation. The missense mutations account for most mutation types. A mutation site with potential clinical significance, R251Q/*, was located in the second Zinc-finger double domain (245–271) (Fig. 4B). Meanwhile, we investigated the correlation between the genetic alterations of MTF1 and the patients’ survival values. We found that the cervical squamous cell carcinoma (CSCC) patients with MTF1 genetic alteration displayed a poor prognosis in DFS (p = 1.405e-3), but not DSS (p = 0.474), OS (p = 0.793) and PFS (p = 0.155) (Fig. 5A). And LIHC patients with MTF1 genetic alteration displayed a poor prognosis in DFS (p = 6.687e-4), DSS (p = 9.466e-3) and PFS (p = 2.001e-3), but not OS (p = 0.134) (Fig. 5B). LUAD patients with MTF1 genetic alteration displayed a poor prognosis in OS (p = 0.0271), but not DFS (p = 0.216), DSS (p = 0.109) and PFS (p = 0.256) (Fig. 5C). Furthermore, STAD patients with MTF1 genetic alteration illustrated a good prognosis in DSS (p = 0.0325) and OS (p = 0.0461), but not DFS (p = 0.334) and PFS (p = 0.0608) (Fig. 5D). According to these findings, we could conclude that the genetic alterations of MTF1 had a strong effect on the prognostic values in the above-mentioned cancers.

### The methylation levels of MTF1 in pan-cancer

**Fig. 6:**
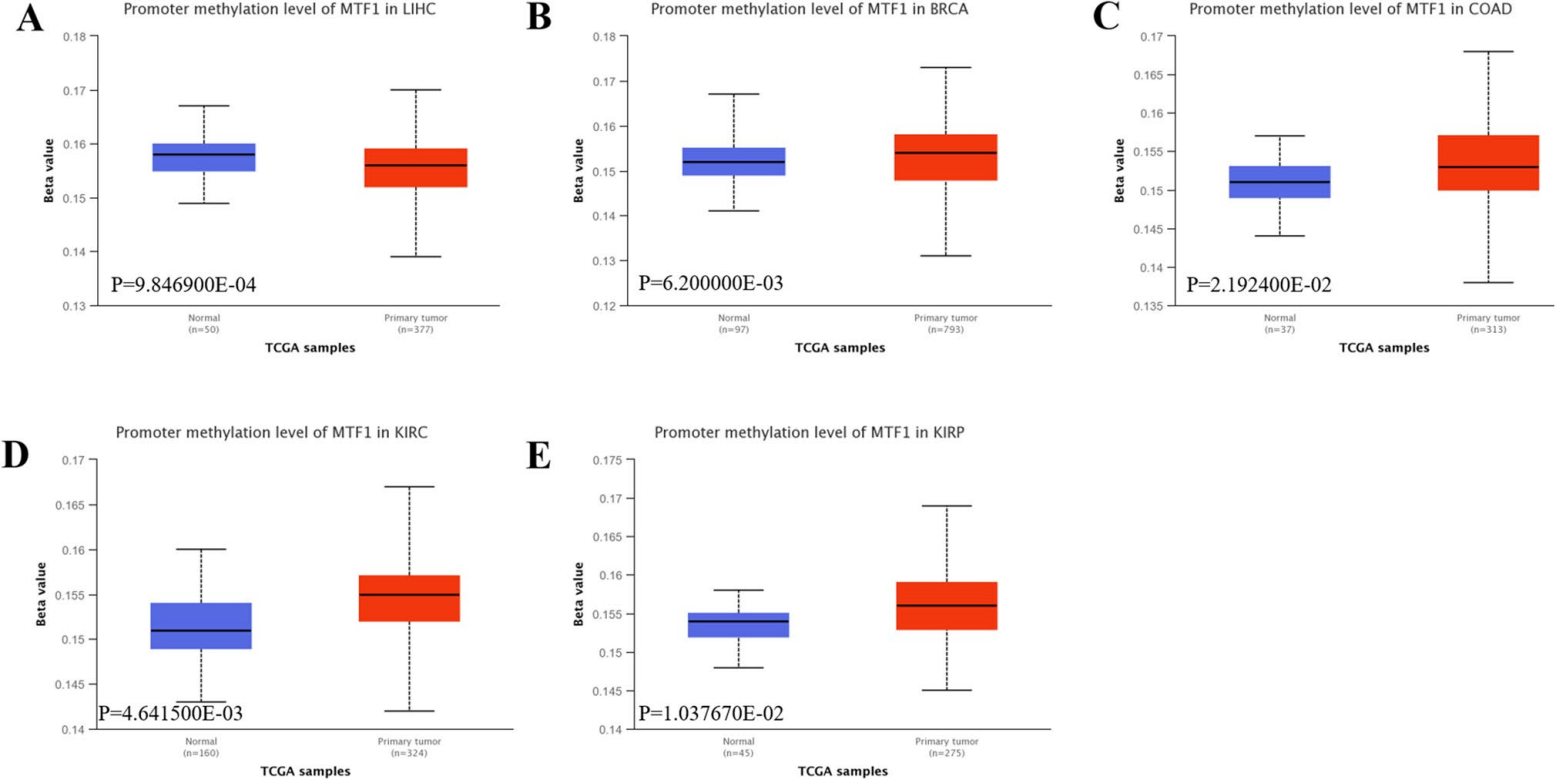
The UALCAN database depicted the methylation levels of MTF1 in pan-cancers. **A**–**E** The diagrams portrayed the promoter methylation levels of MTF1 in patients with LIHC, BRCA, COAD, KIRC and KIRP, respectively (p < 0.05)

Emerging studies have proved the methylation level of cancer-associated genes participate in the progression and treatment of cancers [26]. DNA methylation has been studied to be involved in some cellular processes [27, 28]. DNA methylation has gradually been a diagnostic tool to elevate the accuracy in the pathological diagnosis, especially in the tumors of central nervous system [29]. A report has found that human diseases, aging and cancers were usually along with abnormal DNA methylation levels [30]. As the diagraphs portrayed, the promoter methylation levels of MTF1 in primary tumor group from LIHC (p = 9.846900E-04) was lower than that in the normal groups (Fig. 6A). Moreover, we found that MTF1 methylation levels in the primary tumor group from BRCA (p = 6.200000E-03), COAD (p = 2.192400E-02), KIRC (p = 4.641500E-03), and KIRP (p = 1.037670E-02) were higher than the that in corresponding normal groups (Fig. 6B–E). However, there were no significant changes of MTF1 methylation values in other cancers, such as READ (p = 1.192360E-01), GBM (p = 1.686800E-01), LUSC (p = 2.895400E-01), THCA (p = 4.250800E-01), CHOL (p = 4.342200E-01), STAD (p = 4.353400E-01), pheochromocytoma and paraganglioma (PCPG, p = 6.946400E-01), cervical squamous cell carcinoma and endocervical adenocarcinoma (CESC, p = 3.883200E-01), LUAD (p = 4.760400E-01), PAAD (p = 7.941200E-01), THYM (p = 8.679800E-01), esophageal carcinoma (ESCA, p = 9.065400E-01) and sarcoma (SARC, p = 9.610400E-01) (Supplementary Figure S6A-M). In addition, the promoter methylation levels of MTF1 in primary tumor group from prostate adenocarcinoma (PRAD, p = 1.46826995006677E-12), BLCA (p = 1.017200E-03), head and neck squamous cell carcinoma (HNSC, p = 4.837900E-03) and UCEC (p = 3.52490259203364E-11) were lower than that in the normal groups (Supplementary Figure S6N-Q). Meanwhile, DiseaseMeth version 2.0 also depicted the levels of MTF1 methylation in various cancers (Supplementary Figure S7). Thus, these methylation signatures require further validation in different tumors.

### Identification the effects of MTF1 expression on the immune infiltration cells

**Fig. 7:**
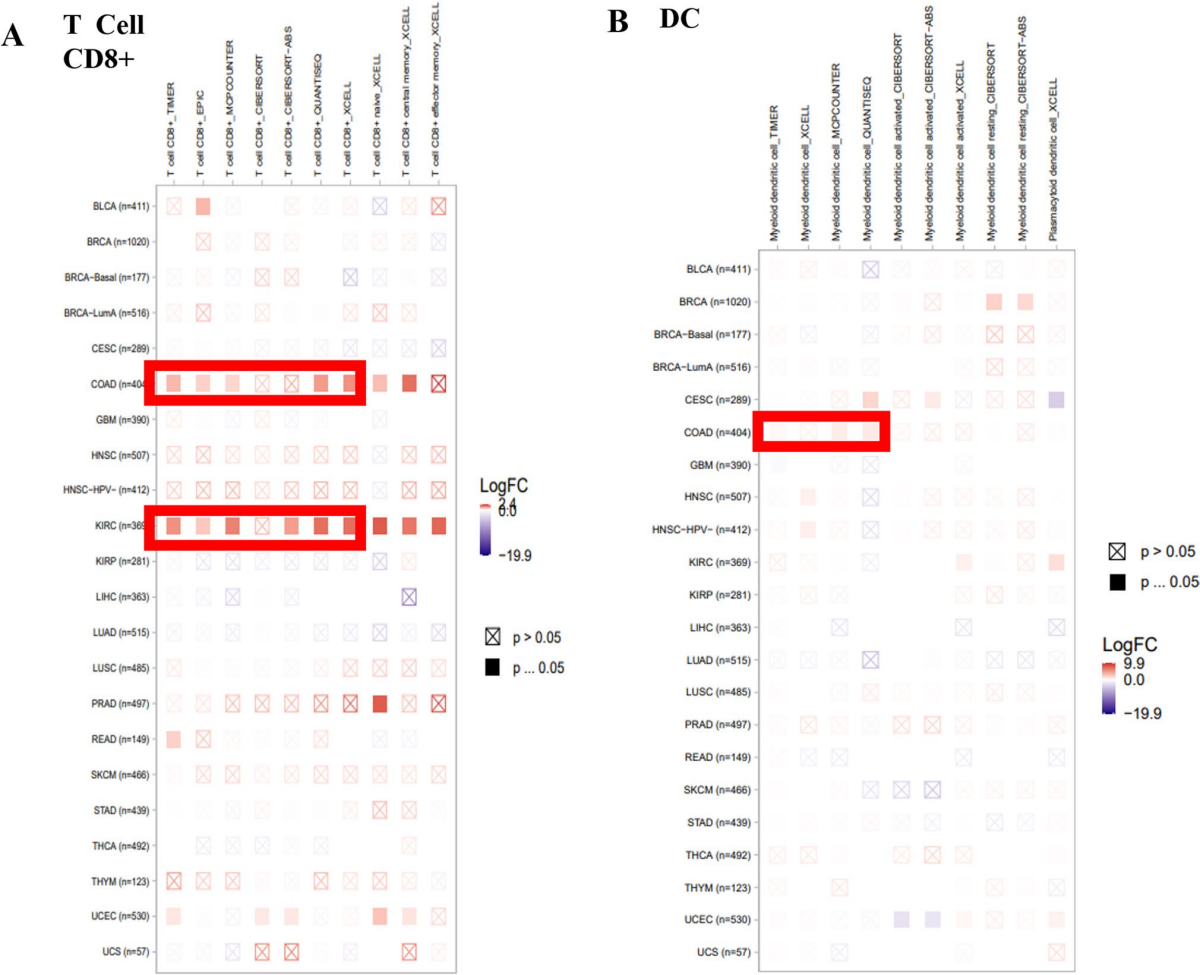
TIMER2.0 database showed the relationship between MTF1 expression and immune cell infiltration. **A**–**B** The correlations between MTF1 expression and immune infiltration of T cell CD8 + and DC were analyzed by some algorithms, including TIMER, EPIC, MCPCOUNTER, CIBERSORT, CIBERSORT-ABS, QUANTISEQ and XCELL

A study has studied the crucial role of cuproptosis-related genes (CRGs) in lung adenocarcinoma and found that MTF1, SLC31A1, FDX1 and DLD were associated with the tumor microenvironment and immune responses in lung adenocarcinoma [31]. Meantime, another study has revealed that CRGs exert great effects in triple-negative breast cancer. And the CRGs were importantly related to the regulation of tumor immunity and prognosis of triple-negative breast cancer [32]. In order to further explore the relationship between the immune cell infiltration and MTF1 expression in TCGA pan-cancer, several algorithms (TIMER, EPIC, MCPCOUNTER, CIBERSORT, CIBERSORT-ABS, QUANTISEQ and XCELL) were employed in TIMER2.0. We concluded that MTF1 expression had a positive relationship with the immune infiltration of T cell CD8 + in COAD and KIRC (Fig. 7A). The expression levels of MTF1 were discovered to possess a positive correlation with DC in COAD (Fig. 7B). Meanwhile, Supplementary Figure S8A-D and Supplementary Figure S9A-C indicated that there were no clear relationships between the MTF1 expression and other immune infiltration cells, including Tregs, CAF, monocyte, neutrophil, B cell, macrophage and NK cell. These results revealed the potential regulatory roles of MTF1 in the immune infiltration of T cell CD8 + and DC cells.

### The expression pattern of MTF1 at single cell level and its correlation with functional status

**Fig. 8:**
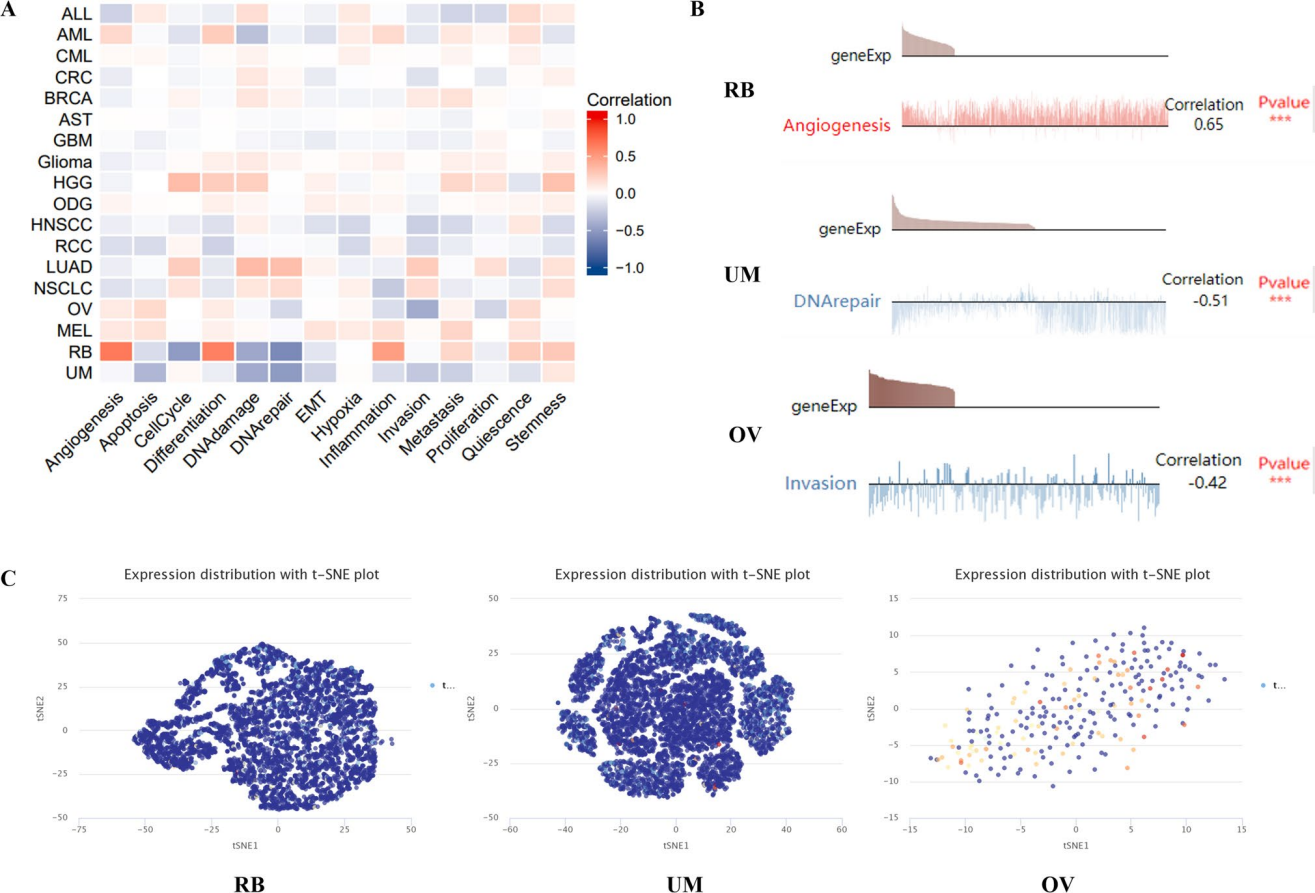
The CancerSEA database portrayed the MTF1 expression at single cell levels. **A**–**B** The correlations between the expression levels of MTF1 and different functional status in pan-cancer. **C** The expression distributions of MTF1 at single cells from RB, UM and OV samples was analyzed by t-SNE diagrams

The CancerSEA database was employed to investigate the expression levels of MTF1 at single cell level in human cancers (Supplementary Table S2). Figure 8A depicted that the expression levels of MTF1 in retinoblastoma (RB) had a positive correlation with angiogenesis and differentiation. Furthermore, MTF1 expression in RB possessed a negative correlation with cell cycle, DNA damage and DNA repair. At the same time, MTF1 expression in uveal melanoma (UM) was strongly negatively related to apoptosis, DNA damage and DNA repair. MTF1 expression in OV possessed a negative correlation with invasion. Meanwhile, the diagraphs illustrated the links between MTF1 expression and angiogenesis in RB, DNA repair in UM and invasion in OV (Fig. 8B). The t-SNE plot showed the distribution of MTF1 in RB, UM and OV at single cell levels (Fig. 8C). According to these results downloaded from the CancerSEA website, we found that MTF1 could be essential in the regulation of cancer-associated biological functions.

### MTF1-associated genes in the metabolism-related pathways

**Fig. 9:**
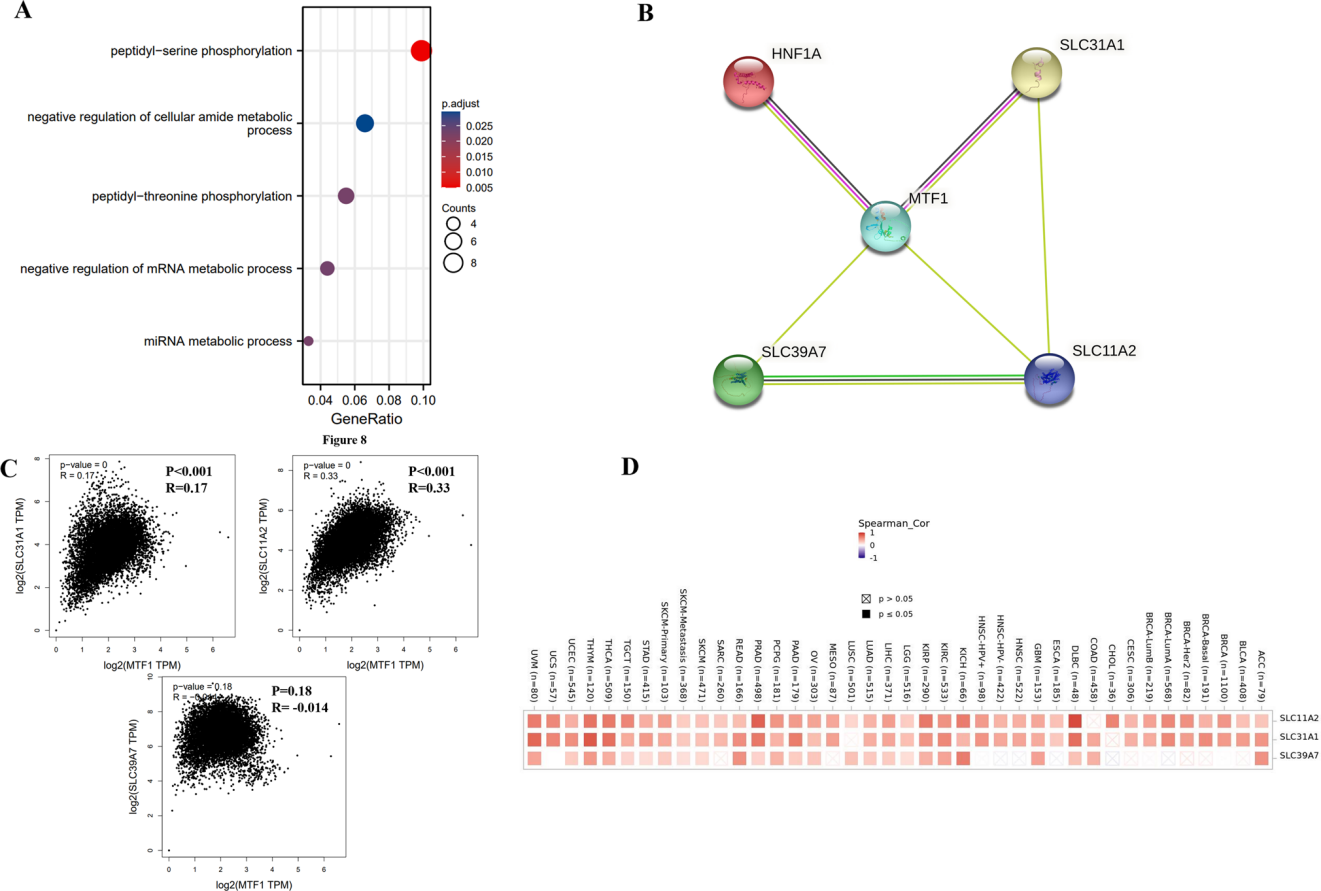
MTF1-associated genes in the metabolism-related pathways. **A** The GO/KEGG analysis of MTF1-associated genes (p < 0.05). **B** The STRING database revealed that MTF1were linked with four energy metabolism-related molecules. **C** The GEPIA2.0 database revealed the correlations between MTF1 expression and three metabolism-related genes (SLC11A2, SLC31A1 and SLC39A7). **D** The heatmap depicted the correlations between MTF1 expression and three genes (SLC11A2, SLC31A1 and SLC39A7)

We conducted functional enrichment of the MTF1-associated genes in cancers. The GEPIA2.0 database was applied to obtain the top 100 MTF1-correlated genes (Supplementary Table S3). GO and KEGG demonstrated that MTF1-associated genes were functionally enriched in several metabolism-related pathways, such as peptidyl-serine phosphorylation, negative regulation of cellular amide metabolic process, peptidyl-threonine phosphorylation, etc. (Fig. 9A). Then, we used the STRING database to investigate the MTF1 interacted energy metabolism-related molecules. And four metabolism molecules were found to be linked with MTF1, including HNF1A, SLC11A2, SLC31A1 and SLC39A7 (Fig. 9B). And we found that MTF1 expression had a positive correlation with the expression levels of SLC31A1 (R = 0.17, p < 0.001), SLC11A2 (R = 0.33, p < 0.001) in pan-cancer (Fig. 9C). Moreover, the heatmap confirmed the positive relationship between MTF1 and SLC31A1 and SLC11A2 in almost all cancer types (Fig. 9D).

### Knockdown of MTF decreased cell growth in LIHC cells

**Fig. 10:**
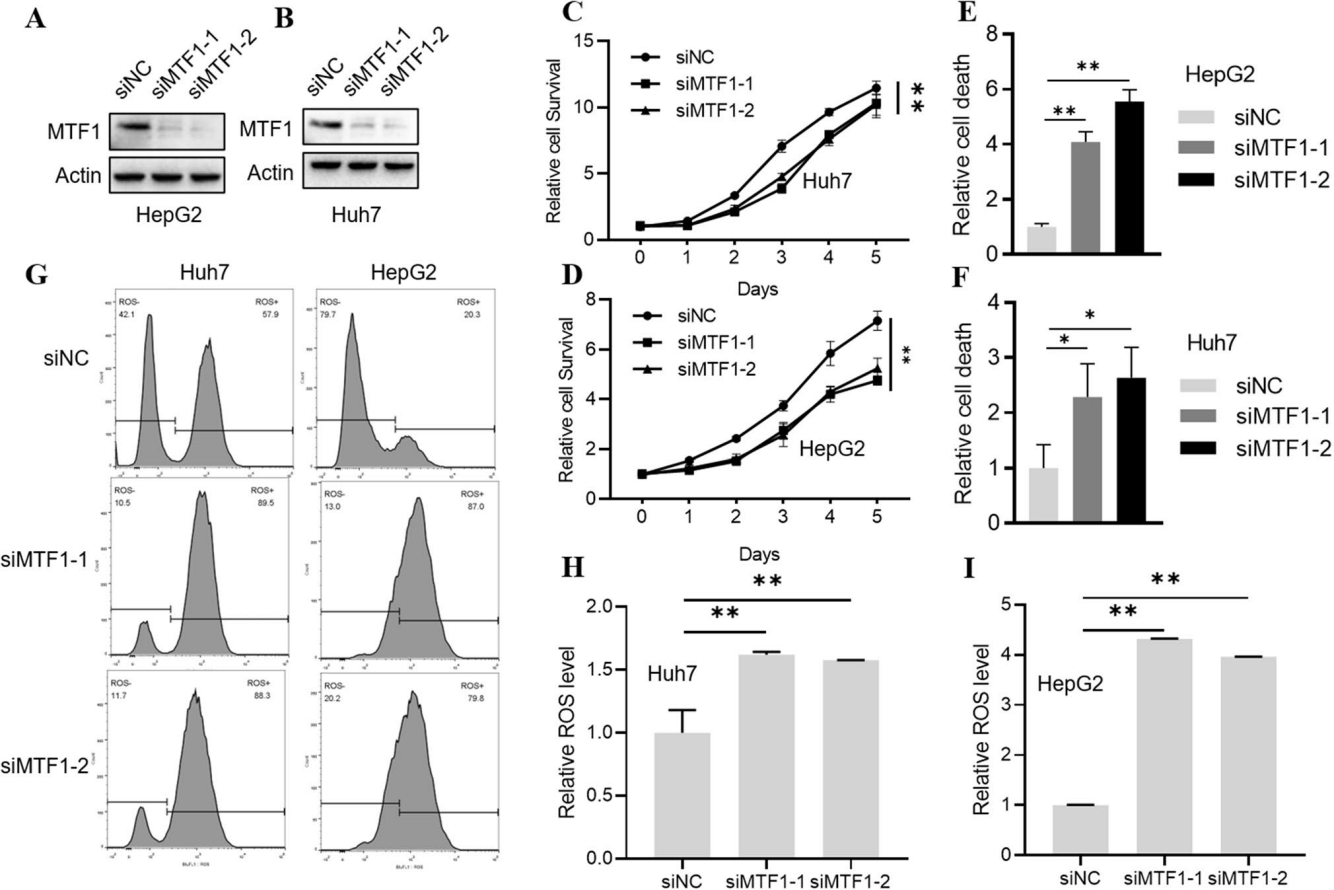
Evaluation of MTF1 knockdown in LIHC cells HepG2 and Huh-7. **A**–**B** Western blot verified the efficiency of siRNA knockdown. **C**–**D** Knockdown of MTF1 suppressed the cell proliferation. **E**–**F** Knockdown of MTF1 induced the cell death. **G**–**I** Knockdown of MTF1 increased the concentration of reactive oxygen species (ROS)

Based on its potential oncogenic roles in LIHC, we evaluated the effect of MTF1 knockdown on the cell growth of LIHC cells (Fig. 10A, B). We found that knockdown of MTF1 could significantly inhibit cell proliferation in two LIHC cell lines HepG2 and Huh7 (Fig. 10C, D). Meanwhile, downregulation of MTF1 could significantly induce cell death in HepG2 and Huh7 cells (Fig. 10E, F). The cellular concentration of reactive oxygen species (ROS) was also upregulated upon MTF1 knockdown (Fig. 10G–I). The findings implicated that the important roles of MTF1 in the induction of cell death in LIHC cells.

## Discussion

Cuproptosis is a distinctive and emerging form of Cu-dependent cell death [33]. There are a lot of evidence highlights the importance effect of cuproptosis in cancer research. Recent studies uncovered that cuproptosis is associated with the tricarboxylic acid (TCA) cycle in human health and disease [34]. Knockdown of the CRGs ferredoxin 1(FDX1) and lipoic acid synthetase (LIAS) results in the cumulation of pyruvate and α-ketoglutarate as well as the consumption of succinate, thereby suppressing the TCA cycle and reducing Cu-induced cell death [35]. Meanwhile, FDX1 has been found to exhibit high expression in multiple cancers, which correlates with better prognosis in KIRC patients [36]. Antioxidant 1 copper chaperone (ATOX1) could promote copper transport via ATP7A-LOX signaling, leading to metastasis of breast cancer cells [37]. Yan et al. has carried out systematic evaluations to predict the CRGs-based prognosis models for LIHC patients [38]. Moreover, some CRGs, including PIH1D2, SLC23A2, SLC25A5, ATP7A, PDHX and COX7B, were reported to be tightly linked with the grading and immune infiltration of esophageal carcinoma [39]. Because of the strong involvement of copper in the cancer pathogenesis, exploring the function of CRGs in cancers has important implications for the potential cancer therapy.

Cuproptosis-associted MTF1 has been verified to participate in the adaptation of cells to some environmental changes, such as exposure to the heavy metals and oxidative stress. MTF1 was found to activate MTs and thus prevent cells from cadmium, zinc, copper and other heavy metals. Moreover, the upregulation level of MTF1 and the downregulation level of XAF1 were investigated in various kinds of tumors, and the alteration of MTF1-XAF1 could cause cancer resistance of apoptosis induced by heavy metal [40, 41]. Inhibiting the MTF1 expression could downregulate the LPS-induced apoptosis of MLE-12 cells. The absence of Methyl-CpG binding domain protein 2 (MBD2) could ameliorate the apoptosis of alveolar epithelial cells through upregulating MTF1 [42]. Moreover, the suppression of mutated in Ataxia-Telangiectasia (ATM) contributed to the nuclear translocation of MTF1, participating in the part of ferroptosis protection [43]. In addition, MTF1 could regulate the targeted gene transcription through recognizing the metal-responsive elements (MREs) [44, 45]. A study has demonstrated that MTF1 could also be coactivated and deacetylated by an epigenetic regulator, sirtuin 6 (SIRT6). MTF1 and SIRT6 could exert synergized effects on the hepatic defense mechanism [46]. Another study has evaluated the expression levels of MTF1 in ovarian cancer samples and found the upregulated expression of MTF1 in ovarian cancer. Knockdown of MTF1 by lentiviral CRISPR/Cas9 could obviously suppress the metastasis of ovarian cancer cells [7]. Mechanistically, a recent study has demonstrated that suppressing ATM could enhance the MTF1 nuclear translocation, which was essential in protecting ferroptosis [43]. Moreover, the MTF1 expression level could be elevated by As2O3, participating in the regulation of lncRNA OTUD6B-AS1 in responding to oxidative stress [47].

To investigate whether MTF1 plays an important role in different types of cancers, we have conducted a pan-caner analysis of MTF1 and evaluated its underlying mechanisms. Using multiple bioinformatics databases, we explored the MTF1 expression across 33 types of tumors based on TCGA database. The expression levels of MTF1 in the tissues of BRCA, COAD, KICH, KIRC, KIRP, LUAD, LUSC, THCA, UCEC, BLCA and READ were lower than the that in corresponding control tissues. And the MTF1 expression in CHOL and LIHC is higher than that in corresponding control tissues. Through the analysis of HPA database, we could find that the protein levels of MTF1 in the normal kidney, testis and colon tissues were higher than that in the corresponding tumor tissues. The GEPIA2.0 database further indicated that the up-regulated MTF1 expression in GBM, LAML, LGG and PAAD. In contrary, the MTF1 expression in ACC, DLBC, OV, TGCT, THYM, UCS and SKCM is lower than that in normal tissues. In addition, GEPIA2.0 database demonstrated that high MTF1 expression was linked with poor OS and DFS in LGG. By contrast, high MTF1 expression was related to the good OS and DFS in KIRC. The Kaplan–Meier plotter portrayed that high MTF1 expression was linked with good OS, FP and PPS in lung cancer, good OS, PPS and PFS in ovarian cancer and good OS, PPS, RFS and DMFS in breast cancer. The above findings have pointed out that MTF1 could be served as a novel prognostic biomarker for cancers.

Single-cell sequencing could implicate the somatic mutations and provide a destination for the exploration of clonal dynamics [48–50]. Also, it could play a vital role in investigating the composition in different cell types [51–53]. The employment of single-cell sequencing in cancer could enhance the understanding of the biological functions of cancer-associated genes [54–56]. The further research about the view of single-cell sequencing will benefit the prognostic prediction and clinical treatment of cancer patients [57–59]. A study has conducted deep single-cell RNA sequencing on T cells obtained from six liver cancer patients and found that exhausted CD8 + T cells and Tregs could enrich and clonally expand in liver cancer [60]. In our study, we applied the CancerSEA to explore MTF1 expression at single cell levels across some cancers. And we found MTF1 in RB was positively associated with angiogenesis. MTF1 expression in UM was negatively associated with DNA repair. Moreover, the MTF1 expression in OV was negatively correlated with invasion. These findings suggested that MTF1 could be essential in the regulation of cancer-associated biological functions.

Cancer cells can alter the tumor immune microenvironment (TIME) through interacting with multiple cells and molecules, thereby affecting the development and treatment of cancer cells [61, 62]. Previous research has shown that different types of immune cells in the TIME have different implications for cancer pathology [63, 64]. DCs, the vital antigen-presenting cells in the immune system, play an essential role in activating the immune response. Recent research has indicated that the anti-tumor function of DCs is usually suppressed in the tumor microenvironment [65]. In addition, high infiltration degree of selected immune cells (especially cytotoxic T cells) in TIME can predict the prognosis of cancer patients [66]. A study has shown that the cuproptosis-related genes LIAS, PDK1 and BCL2L1 were strongly related to immune cells infiltrating in osteosarcoma, serving as the reliable targets for predicting the patients’ immune response [67]. Here, we used several algorithms from TIMER2.0, such as TIMER, EPIC, MCPCOUNTER, CIBERSORT, CIBERSORT-ABS, QUANTISEQ and XCELL, to analyze the relationship between immune cell infiltration and MTF1 expression in pan-cancer. We found that the positive correlation between MTF1 expression and T cell CD8 + immune infiltration in COAD and KIRC. Additionally, MTF1 expression levels were positively correlated with DC in COAD. Correlation analysis proved the potential regulation roles of MTF1 in the infiltration of T cell CD8 + and DC cells. However, the mechanism of MTF1 on the regulation of immune cell infiltration remains to be further explored.

To sum up, through a series of pan-cancer analysis, we investigated the expression, prognosis, genetic alteration and methylation profiles of MTF1 in various cancers. Moreover, the MTF1 expression at single cell levels and the functional signaling pathways were also explored. These findings have clarified that MTF1 plays an essential role in the cancer progression, cancer metabolism and immune regulation. And this article would provide a new strategy for the MTF1-based survival prediction in several cancer patients. However, some limitations still exist in our research. Our results were mainly derived from the bioinformatics analysis and preliminary cellular experiments. Bioinformatics still limited by data quality, individual case variation and lack of temporal dynamics. The complexity of data interpretation, the paucity of currently knowledge and incomplete databases will also require more advanced algorithms and technological progress in the future. In addition, the diverse expression values and prognostic profiles in multiple cancers predicts the highly complex roles of MTF1 in human cancers. Thus, the underlying molecular mechanism of MTF1 should be further evaluated to demonstrate the functional roles of MTF1 in cancers.

## Supplementary Information

Supplementary file 1: Figure S1. The expression levels of MTF1 in some types of cancers.GEO database showing the MTF1 expression in tumors and the corresponding normal tissues, such asadrenocortical carcinoma,colon cancer,kidney cancer,liver cancer andglioma.

Supplementary file 2: Figure S2. The effects of MTF1 on the pathological stages in cancer patients.The GEPIA2.0 database portrayed the effects of MTF1 expression on the pathological stages of patients with several cancers, such as ACC, BLCA, BRCA, CESC, CHOL, COAD, DLBC, ESCA, KICH, KIRP, LUAD, LUSC, PAAD, READ, STAD, SKCM, TGCT, THCA, UCEC and UCS

Supplementary file 3: Figure S3. The CPTAC from Ualcan database showed MTF1 protein levels in multiple types of cancers. This diagraph depicted the protein levels of MTF1 in UCEC, lung cancer, glioblastoma, head and neck cancer

Supplementary file 4: Figure S4. The prognostic values of MTF1 expression in four cancers.The Kaplan-Meier plotter database showed the effects of MTF1 expression on the survival valuesin some types of cancers, includinglung cancer,ovarian cancer,breast cancer andliver cancer

Supplementary file 5: Figure S5. The prognostic values of MTF1 expression in three cancers.The Kaplan-Meier plotter database displayed the effects of MTF1 expression on the overall survivalin three cancers with radiotherapy or chemotherapy, includinglung cancer,ovarian cancer andbreast cancer

Supplementary file 6: Figure S6. The UALCAN database illustrated the MTF1 methylation levels in several cancers.The diagraphs demonstrated the promoter methylation levels of MTF1 in READ, GBM, LUSC, THCA, CHOL, STAD, PCPG, CESC, LUAD, PAAD, THYM, ESCA, SARC PRAD, BLCA, HNSC and UCEC respectively

Supplementary file 7: Figure S7. DiseaseMeth version 2.0 depicted the levels of MTF1 methylation in various cancers. The pictures showed the promoter methylation levels of MTF1 in READ, COAD, PA, OV, BLCA, Meningiomas, OSCC, KICH, PAAD, PRAD, GCC, HNSC, UCEC, TGCT, KIRP and KIRC respectively

Supplementary file 8: Figure S8. TIMER2.0 database showed the relationship between MTF1 expression and immune cell infiltration.The correlations between MTF1 expression and immune infiltration of Tregs, CAF, monocyte and neutrophil were analyzed by some algorithms

Supplementary file 9: Figure S9. TIMER2.0 database showed the relationship between MTF1 expression and immune cell infiltration.The correlations between MTF1 expression and immune infiltration of B cell, macrophage and NK cells were analyzed by some algorithms

Supplementary file 10: Table S1. The bioinformatics platforms that are used for exploring the functions of MTF1

Supplementary file 11: Table S2. The correlation between MTF1 and pan-cancers via CancerSEA database

Supplementary file 12: Table S3. The top 100 MTF1-correlated genes that obtained from the GEPIA2.0 database

## Data Availability

All relevant data are within the manuscript and its Supporting Information files.
