# Supplementary figures and images for: The biological significance of cuproptosis-key gene MTF1 in pan-cancer and its inhibitory effects on ROS-mediated cell death of liver hepatocellular carcinoma
Source: Discov Oncol. 2023 Jun 28;14:113. doi: 10.1007/s12672-023-00738-8 (PMC10307746; doi:10.1007/s12672-023-00738-8)

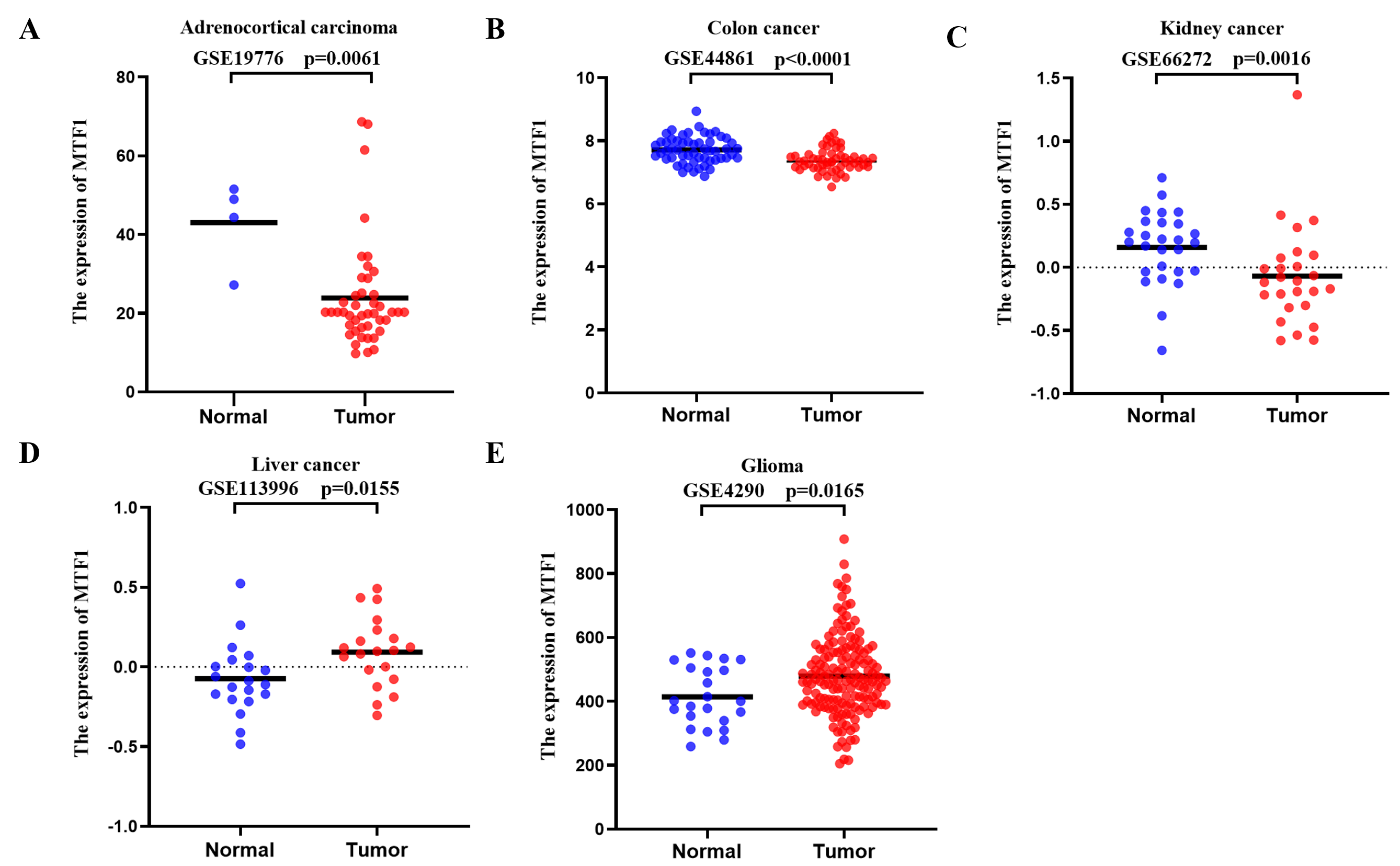

Supplement: Supplementary file 1 — Figure S1. The expression levels of MTF1 in some types of cancers.GEO database showing the MTF1 expression in tumors and the corresponding normal tissues, such asadrenocortical carcinoma,colon cancer,kidney cancer,liver cancer andglioma. [file 12672_2023_738_MOESM1_ESM.tif]

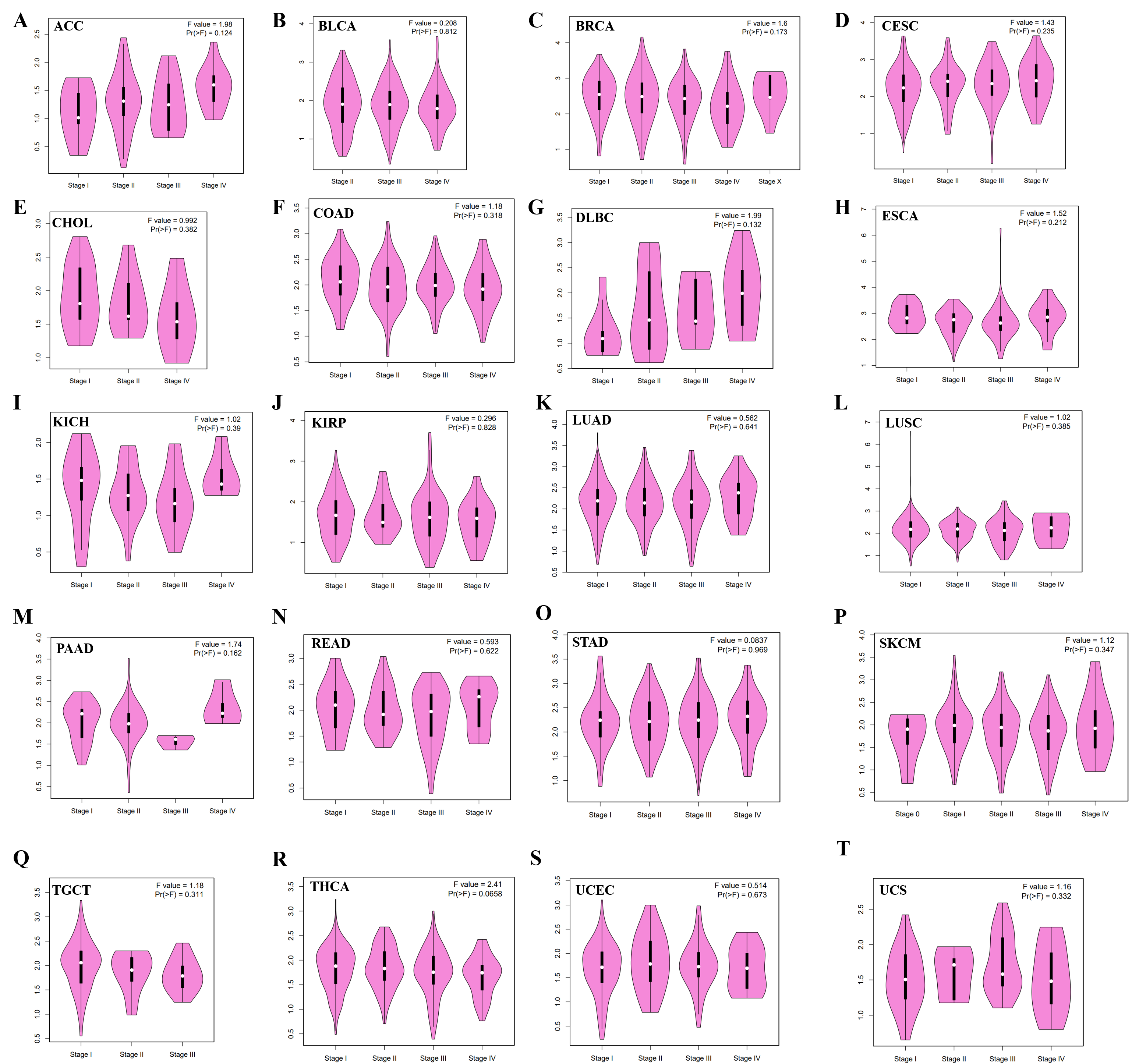

Supplement: Supplementary file 2 — Figure S2. The effects of MTF1 on the pathological stages in cancer patients.The GEPIA2.0 database portrayed the effects of MTF1 expression on the pathological stages of patients with several cancers, such as ACC, BLCA, BRCA, CESC, CHOL, COAD, DLBC, ESCA, KICH, KIRP, LUAD, LUSC, PAAD, READ, STAD, SKCM, TGCT, THCA, UCEC and UCS [file 12672_2023_738_MOESM2_ESM.tif]

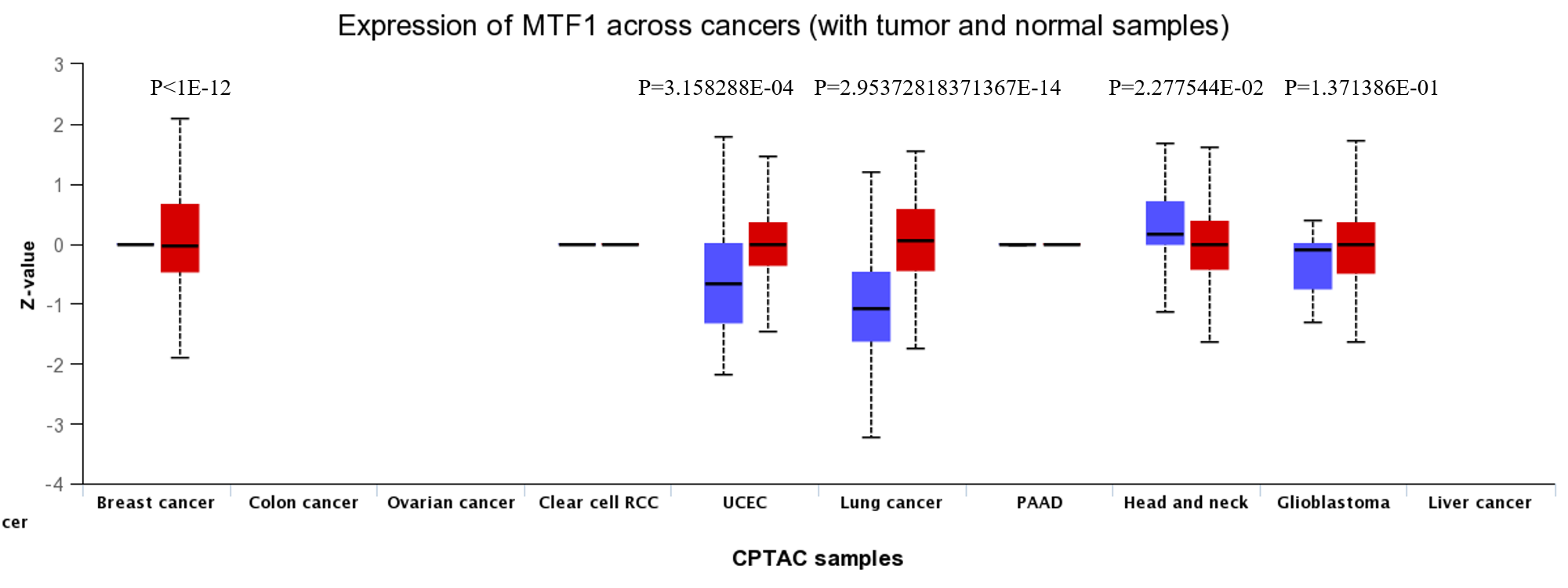

Supplement: Supplementary file 3 — Figure S3. The CPTAC from Ualcan database showed MTF1 protein levels in multiple types of cancers. This diagraph depicted the protein levels of MTF1 in UCEC, lung cancer, glioblastoma, head and neck cancer [file 12672_2023_738_MOESM3_ESM.tif]

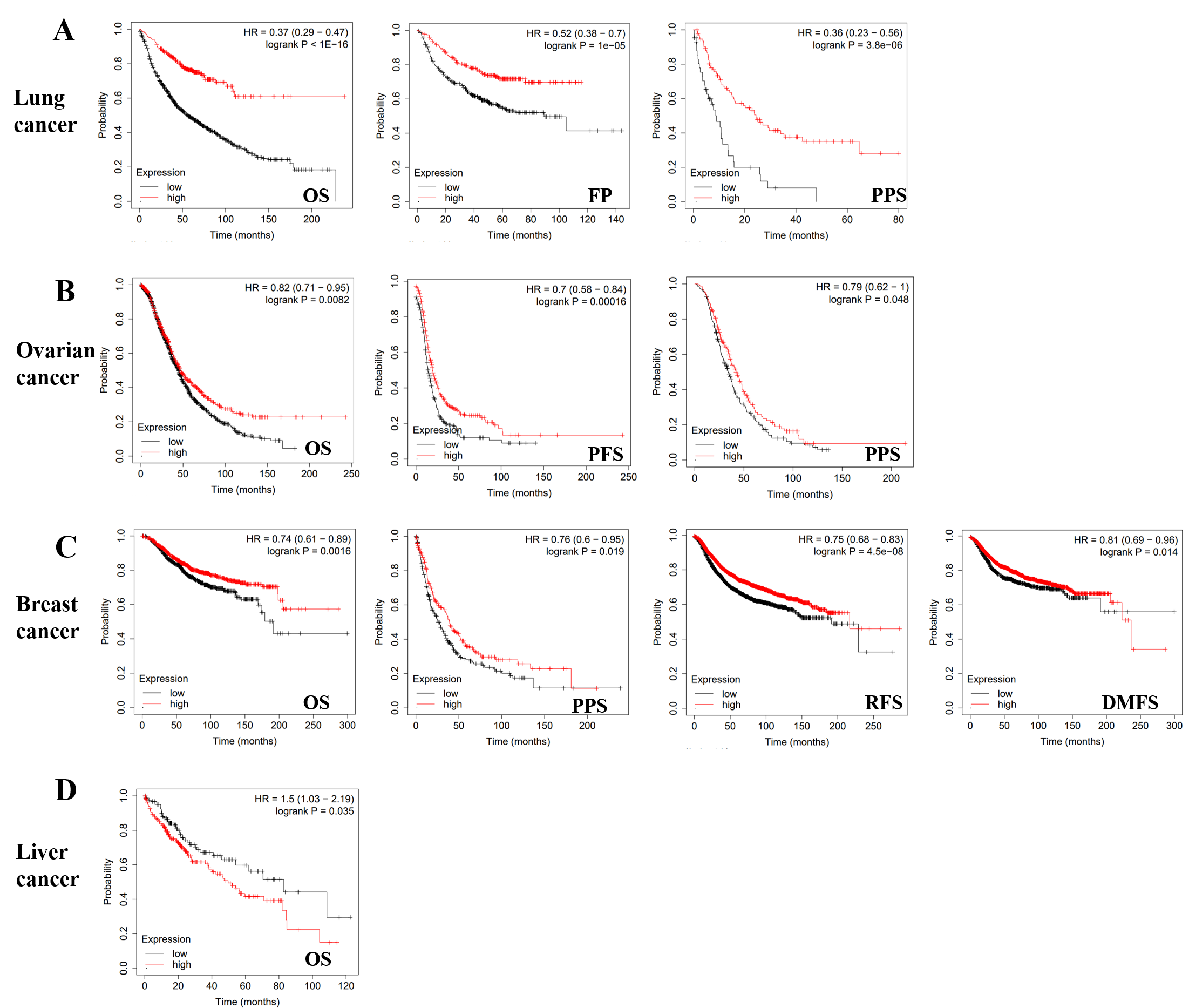

Supplement: Supplementary file 4 — Figure S4. The prognostic values of MTF1 expression in four cancers.The Kaplan-Meier plotter database showed the effects of MTF1 expression on the survival valuesin some types of cancers, includinglung cancer,ovarian cancer,breast cancer andliver cancer [file 12672_2023_738_MOESM4_ESM.tif]

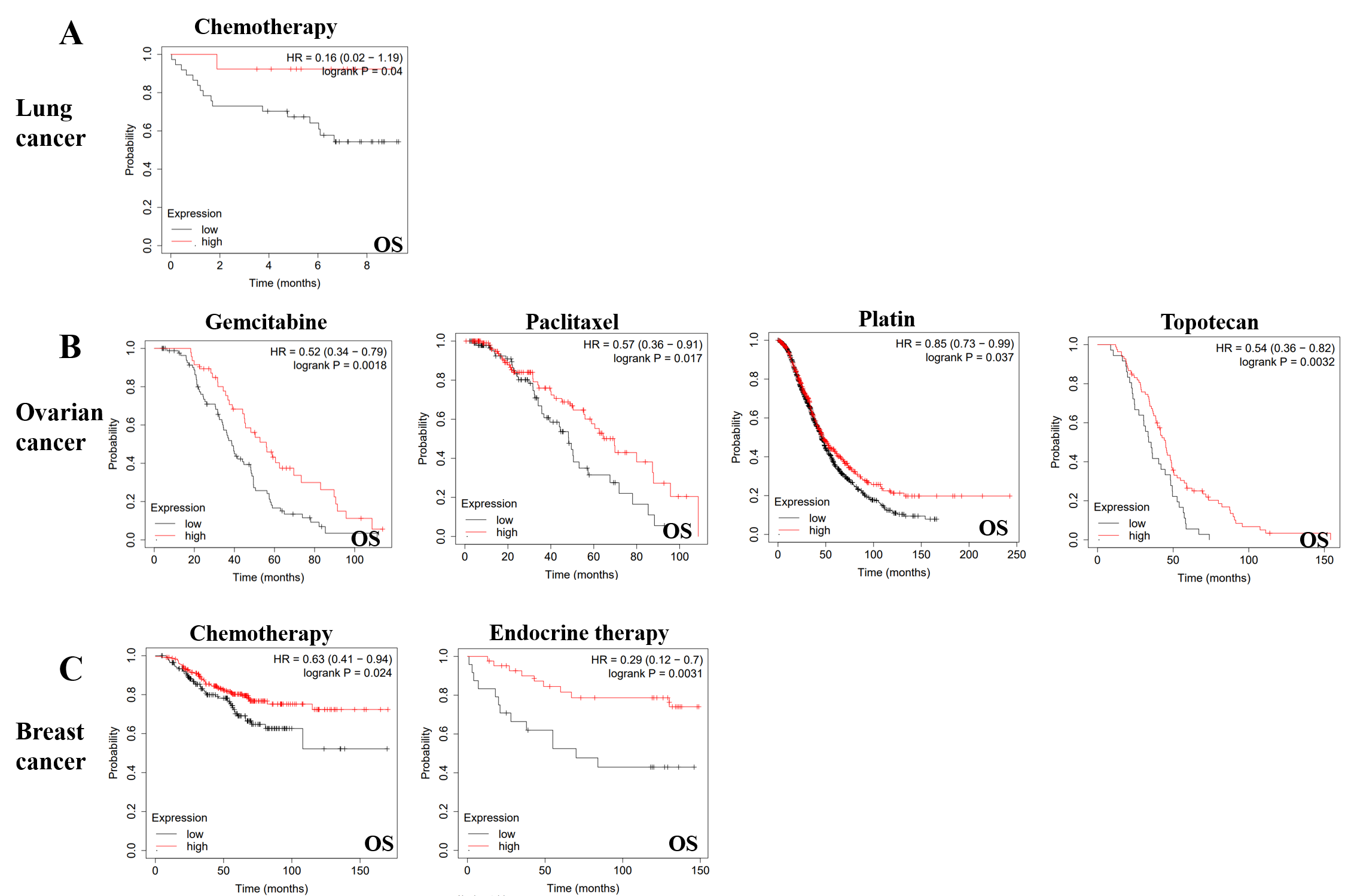

Supplement: Supplementary file 5 — Figure S5. The prognostic values of MTF1 expression in three cancers.The Kaplan-Meier plotter database displayed the effects of MTF1 expression on the overall survivalin three cancers with radiotherapy or chemotherapy, includinglung cancer,ovarian cancer andbreast cancer [file 12672_2023_738_MOESM5_ESM.tif]

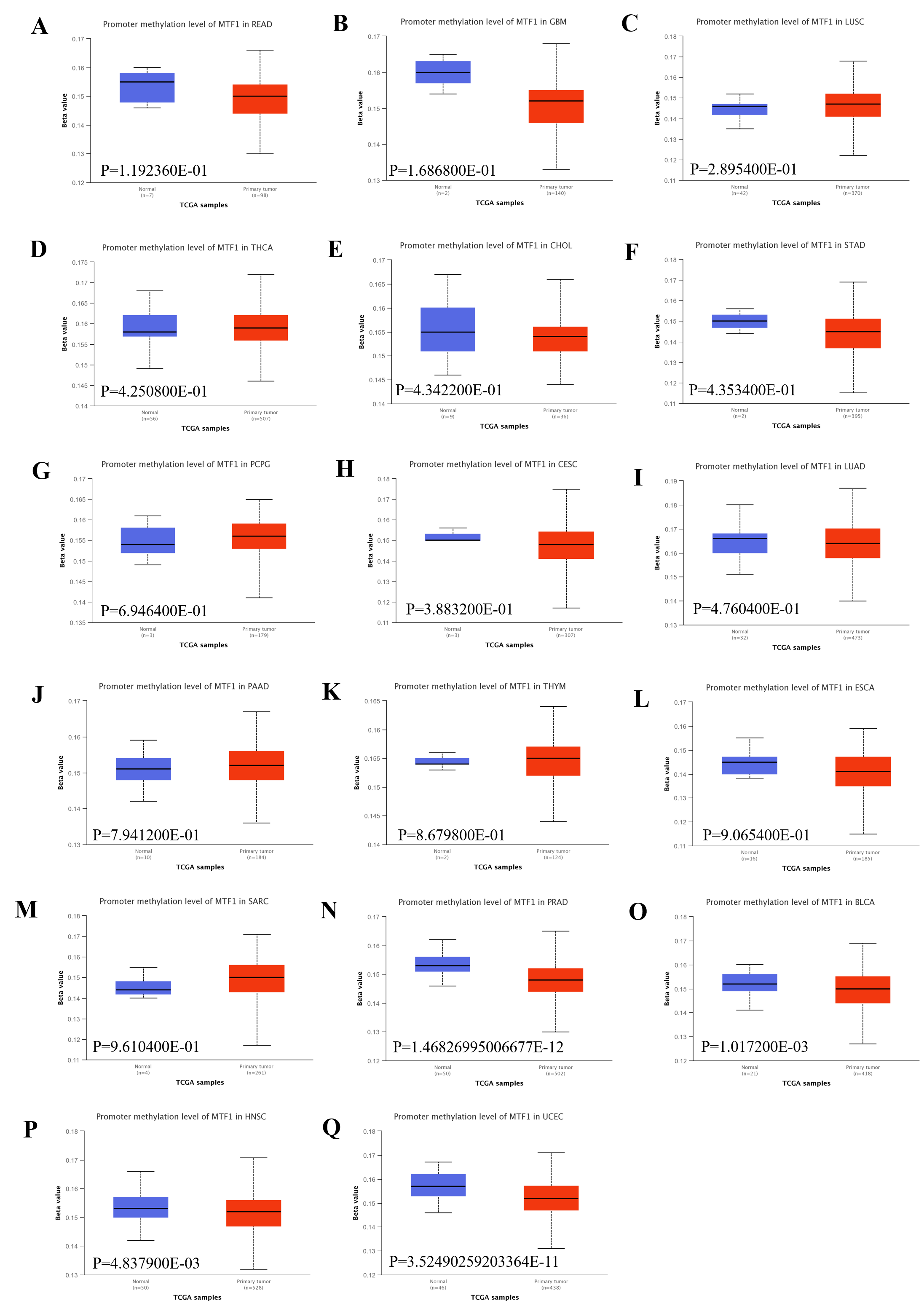

Supplement: Supplementary file 6 — Figure S6. The UALCAN database illustrated the MTF1 methylation levels in several cancers.The diagraphs demonstrated the promoter methylation levels of MTF1 in READ, GBM, LUSC, THCA, CHOL, STAD, PCPG, CESC, LUAD, PAAD, THYM, ESCA, SARC PRAD, BLCA, HNSC and UCEC respectively [file 12672_2023_738_MOESM6_ESM.tif]

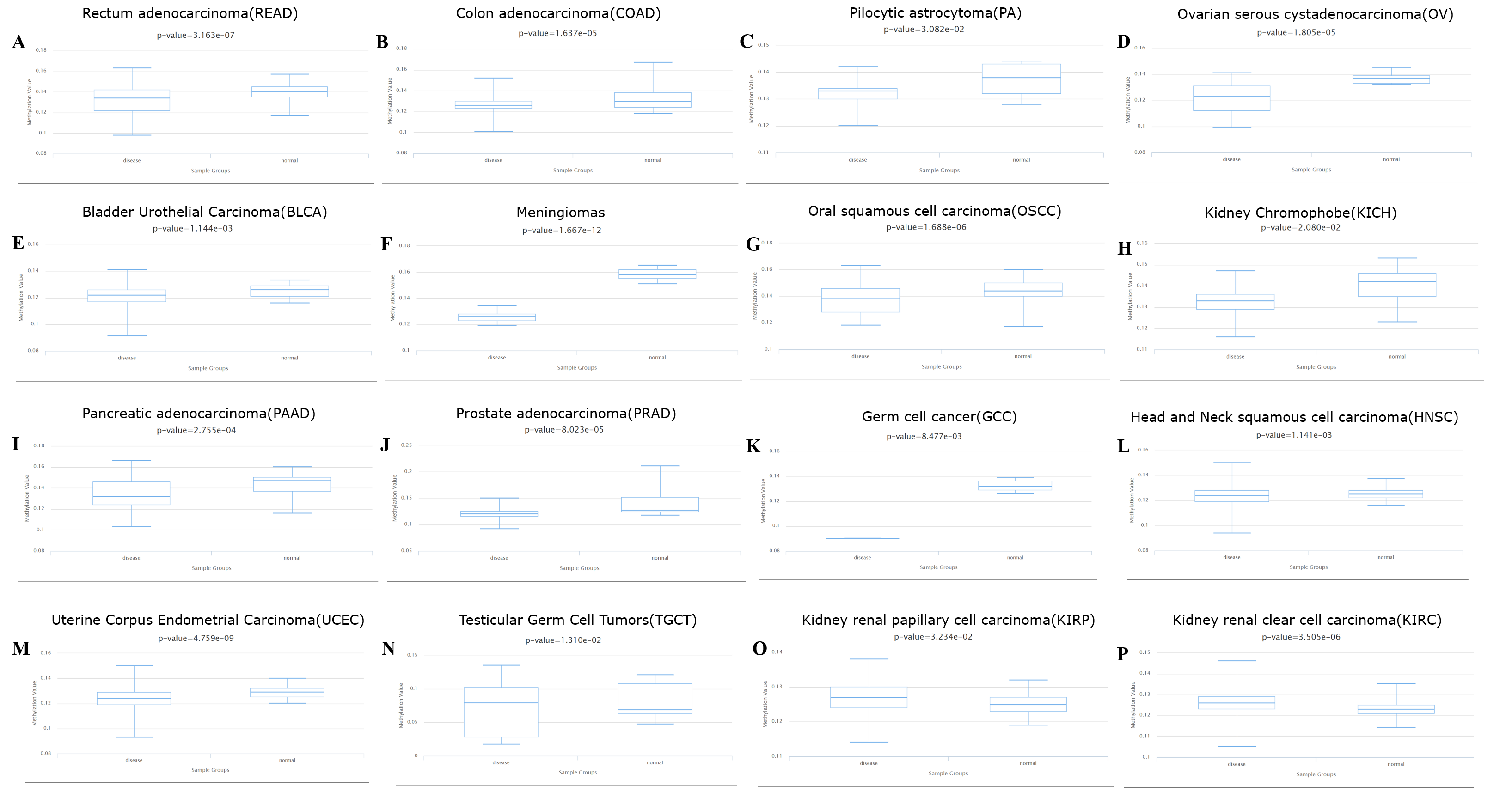

Supplement: Supplementary file 7 — Figure S7. DiseaseMeth version 2.0 depicted the levels of MTF1 methylation in various cancers. The pictures showed the promoter methylation levels of MTF1 in READ, COAD, PA, OV, BLCA, Meningiomas, OSCC, KICH, PAAD, PRAD, GCC, HNSC, UCEC, TGCT, KIRP and KIRC respectively [file 12672_2023_738_MOESM7_ESM.tif]

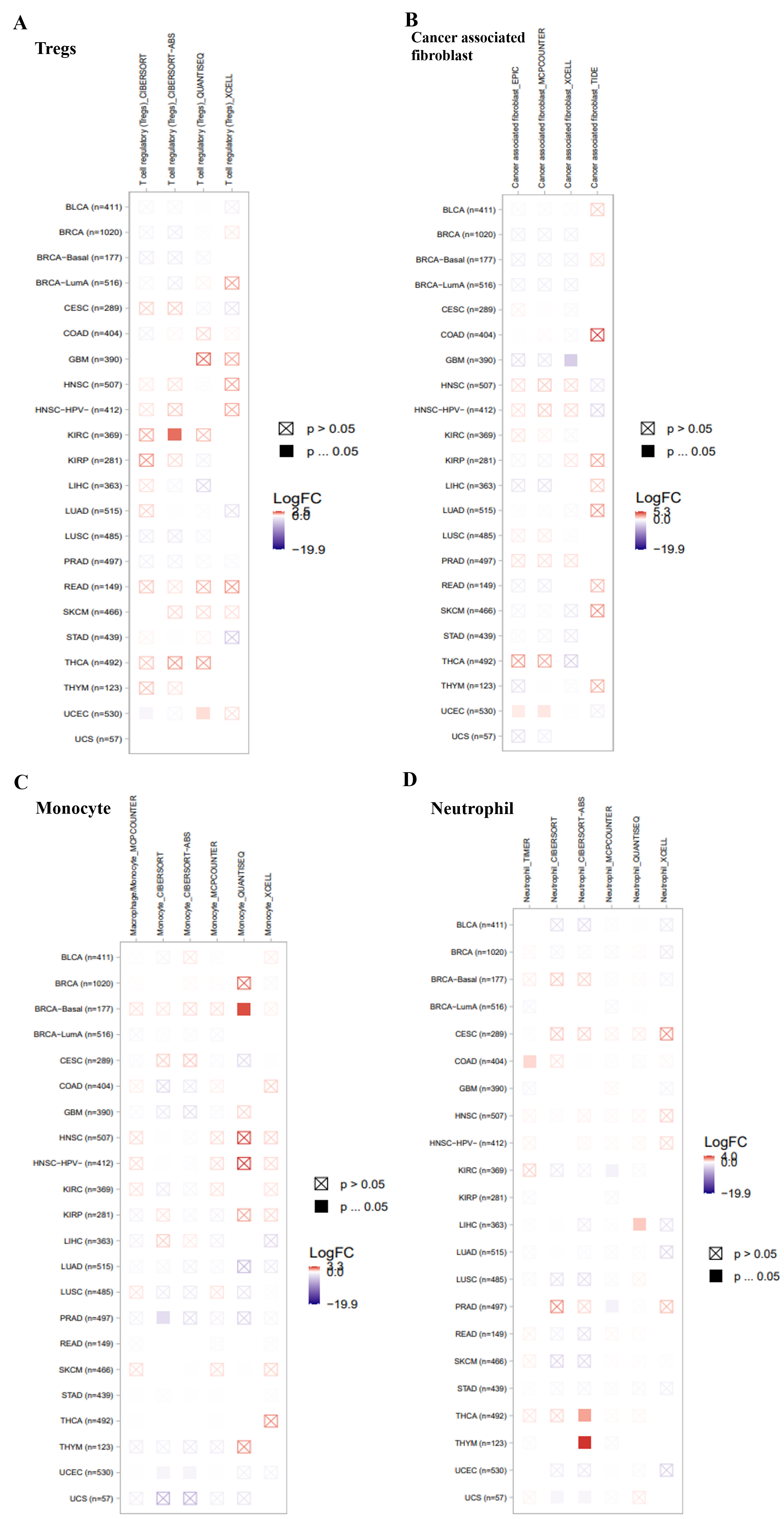

Supplement: Supplementary file 8 — Figure S8. TIMER2.0 database showed the relationship between MTF1 expression and immune cell infiltration.The correlations between MTF1 expression and immune infiltration of Tregs, CAF, monocyte and neutrophil were analyzed by some algorithms [file 12672_2023_738_MOESM8_ESM.tif]

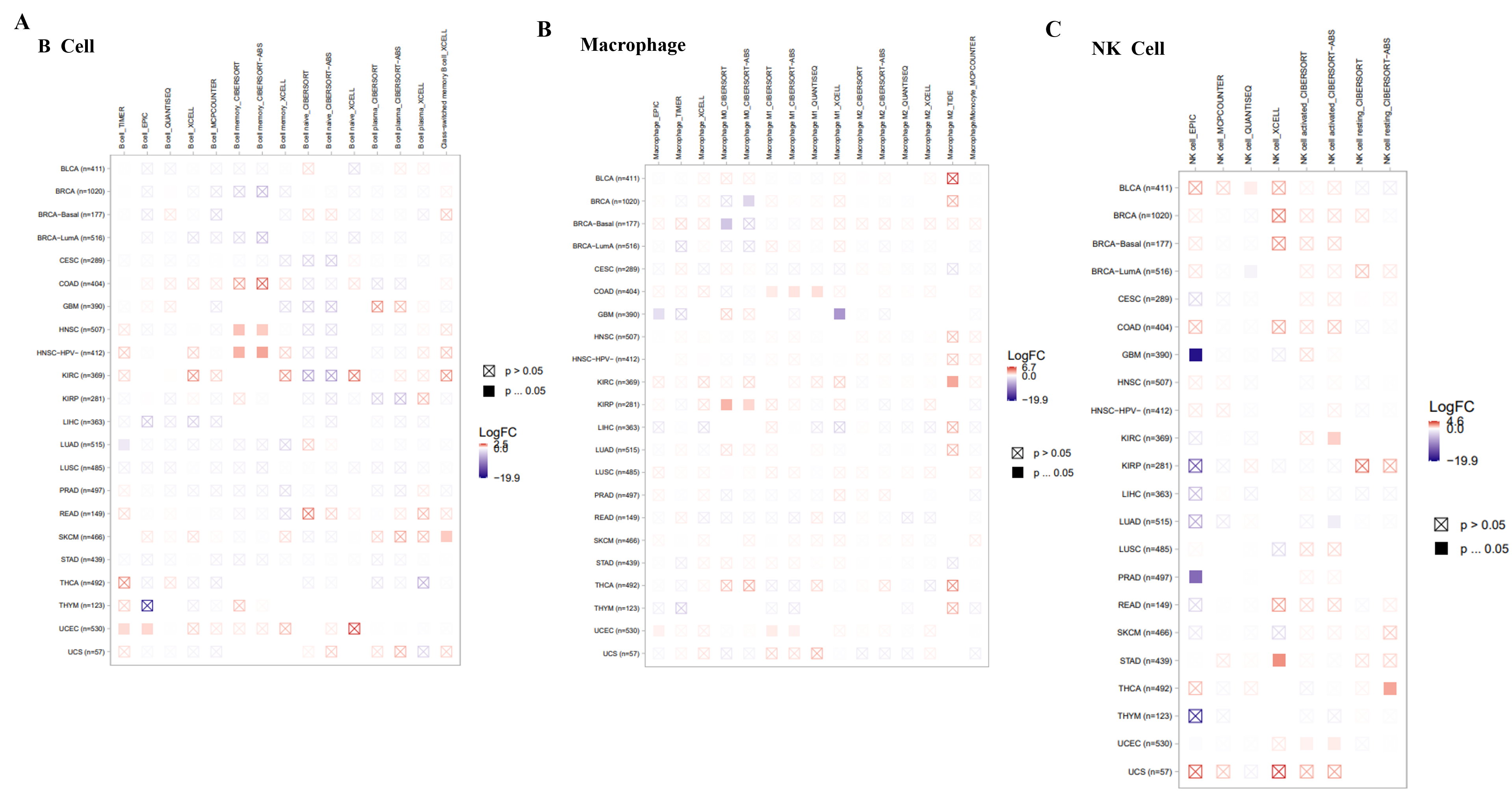

Supplement: Supplementary file 9 — Figure S9. TIMER2.0 database showed the relationship between MTF1 expression and immune cell infiltration.The correlations between MTF1 expression and immune infiltration of B cell, macrophage and NK cells were analyzed by some algorithms [file 12672_2023_738_MOESM9_ESM.tif]
