# Supplementary material for: The biological significance of cuproptosis-key gene MTF1 in pan-cancer and its inhibitory effects on ROS-mediated cell death of liver hepatocellular carcinoma
Source: Discov Oncol. 2023 Jun 28;14:113. doi: 10.1007/s12672-023-00738-8 (PMC10307746; doi:10.1007/s12672-023-00738-8)
Supplement: Supplementary file 10 — Table S1. The bioinformatics platforms that are used for exploring the functions of MTF1 [file 12672_2023_738_MOESM10_ESM.docx]

**Supplementary Table S1. The bioinformatics platforms that are used for exploring the functions of MTF1.**

| **Databases** | **URL** | **References** |
| --- | --- | --- |
| TIMER2.0 | http://timer.cistrome.org/ | [9] |
| TNMplot | https://tnmplot.com/analysis/ | [10] |
| GEPIA2.0 | http://gepia2.cancer-pku.cn/#index | [11] |
| UALCAN | http://ualcan.path.uab.edu/analysisprot.html | [12] |
| HPA | http://www.proteinatlas.org | [13] |
| cBioPortal | https://www.cbioportal.org/ | [14] |
| CancerSEA | http://biocc.hrbmu.edu.cn/CancerSEA/home.jsp | [18] |
| STRING | http://string-db.org | [19] |
| Kaplan-Meier plotter | http://kmplot.com/analysis/ | [22] |
