# Supplementary material for: The biological significance of cuproptosis-key gene MTF1 in pan-cancer and its inhibitory effects on ROS-mediated cell death of liver hepatocellular carcinoma
Source: Discov Oncol. 2023 Jun 28;14:113. doi: 10.1007/s12672-023-00738-8 (PMC10307746; doi:10.1007/s12672-023-00738-8)
Supplement: Supplementary file 11 — Table S2. The correlation between MTF1 and pan-cancers via CancerSEA database [file 12672_2023_738_MOESM11_ESM.docx]

| **Supplementary Table S2. The correlation between MTF1 and pan-cancers via CancerSEA database.** | | | | | | | | | | | | | | |
| --- | --- | --- | --- | --- | --- | --- | --- | --- | --- | --- | --- | --- | --- | --- |
| Cancer | Angiogenesis | Apoptosis | CellCycle | Differentiation | DNAdamage | DNArepair | EMT | Hypoxia | Inflammation | Invasion | Metastasis | Proliferation | Quiescence | Stemness |
| ALL | -0.224 | 0.107 | -0.05 | -0.084 | 0.165 | -0.018 | -0.065 | 0.108 | -0.016 | -0.113 | -0.212 | -0.195 | 0.183 | 0.103 |
| AML | 0.19 | -0.023 | -0.146 | 0.253 | -0.311 | -0.07 | -0.154 | 0.096 | 0.188 | -0.119 | 0.116 | 0.044 | 0.156 | -0.132 |
| CML | 0.026 | 0.036 | -0.04 | 0.019 | 0.059 | 0.011 | 0.004 | 0.072 | 0.014 | 0.008 | 0.049 | -0.001 | 0.069 | -0.022 |
| CRC | -0.097 | 0 | -0.05 | -0.025 | 0.119 | 0.033 | -0.098 | -0.011 | 0.004 | -0.143 | 0.001 | -0.099 | 0.018 | 0.067 |
| BRCA | -0.07 | -0.011 | 0.056 | -0.024 | 0.125 | 0.059 | -0.052 | -0.06 | -0.056 | 0.109 | 0.145 | 0.025 | -0.014 | 0 |
| AST | 0.007 | 0.005 | -0.032 | 0.003 | -0.023 | -0.03 | -0.018 | 0.007 | 0.02 | -0.025 | -0.064 | 0.007 | -0.026 | 0.05 |
| GBM | -0.023 | -0.074 | -0.025 | -0.006 | -0.042 | -0.057 | -0.094 | -0.04 | -0.04 | -0.078 | -0.066 | 0.048 | 0.001 | -0.052 |
| Glioma | -0.077 | -0.042 | 0.018 | 0.078 | 0.104 | 0.05 | 0.014 | 0.042 | 0.069 | 0.013 | 0.076 | 0.067 | 0.012 | 0.08 |
| HGG | -0.057 | -0.001 | 0.334 | 0.25 | 0.239 | -0.006 | 0.068 | -0.043 | 0.026 | -0.036 | 0.198 | 0.142 | -0.13 | 0.307 |
| ODG | 0.049 | 0.013 | 0.007 | 0.077 | 0.047 | -0.015 | 0.074 | 0.059 | 0.046 | -0.065 | 0.019 | 0.038 | 0.044 | 0.074 |
| HNSCC | -0.081 | -0.036 | -0.096 | -0.151 | 0.075 | -0.048 | -0.153 | -0.198 | -0.021 | -0.239 | -0.187 | -0.062 | 0.116 | -0.181 |
| RCC | -0.162 | -0.176 | 0.044 | -0.222 | -0.029 | -0.02 | -0.03 | -0.194 | 0.059 | -0.186 | -0.094 | -0.028 | -0.051 | -0.13 |
| LUAD | -0.088 | -0.021 | 0.25 | -0.108 | 0.341 | 0.32 | 0.059 | -0.069 | -0.078 | 0.258 | -0.018 | 0.162 | -0.146 | 0.144 |
| NSCLC | -0.146 | -0.105 | 0.142 | -0.124 | 0.128 | 0.174 | -0.014 | 0.074 | -0.262 | 0.18 | -0.096 | -0.039 | -0.095 | 0.168 |
| OV | 0.106 | 0.189 | 0.001 | 0.098 | -0.032 | -0.19 | 0.003 | 0.03 | -0.169 | -0.421 | 0.061 | -0.21 | 0.177 | 0.011 |
| MEL | 0.115 | 0.149 | -0.009 | 0.072 | 0.028 | -0.027 | 0.137 | 0.103 | 0.158 | 0.027 | 0.205 | 0.002 | 0.134 | -0.023 |
| RB | 0.654 | -0.172 | -0.507 | 0.622 | -0.443 | -0.6 | -0.127 | -0.003 | 0.485 | -0.023 | 0.198 | -0.083 | 0.253 | 0.271 |
| UM | -0.037 | -0.362 | 0.038 | -0.09 | -0.429 | -0.51 | -0.219 | 0.011 | -0.171 | -0.272 | -0.235 | -0.06 | -0.143 | 0.123 |
