# Supplementary material for: The biological significance of cuproptosis-key gene MTF1 in pan-cancer and its inhibitory effects on ROS-mediated cell death of liver hepatocellular carcinoma
Source: Discov Oncol. 2023 Jun 28;14:113. doi: 10.1007/s12672-023-00738-8 (PMC10307746; doi:10.1007/s12672-023-00738-8)
Supplement: Supplementary file 12 — Table S3. The top 100 MTF1-correlated genes that obtained from the GEPIA2.0 database [file 12672_2023_738_MOESM12_ESM.docx]

| **Supplementary Table S3. The top 100 MTF1-correlated genes that obtained from the GEPIA2.0 database.** | | |
| --- | --- | --- |
| **Gene Symbol** | **Gene ID** | **PCC** |
| SF3A3 | ENSG00000183431.11 | 0.71 |
| SNIP1 | ENSG00000163877.9 | 0.71 |
| RLF | ENSG00000117000.8 | 0.67 |
| KPNA6 | ENSG00000025800.13 | 0.66 |
| CELF1 | ENSG00000149187.17 | 0.65 |
| PUM1 | ENSG00000134644.15 | 0.64 |
| INPP5B | ENSG00000204084.12 | 0.64 |
| GAPVD1 | ENSG00000165219.21 | 0.63 |
| CREB1 | ENSG00000118260.14 | 0.63 |
| DDX3X | ENSG00000215301.9 | 0.63 |
| C1orf109 | ENSG00000116922.14 | 0.63 |
| CSNK1G1 | ENSG00000169118.15 | 0.63 |
| TRIP12 | ENSG00000153827.13 | 0.63 |
| ASXL2 | ENSG00000143970.16 | 0.62 |
| ZBTB11 | ENSG00000066422.4 | 0.62 |
| STRN | ENSG00000115808.11 | 0.62 |
| GMEB1 | ENSG00000162419.12 | 0.62 |
| AGO1 | ENSG00000092847.10 | 0.62 |
| PKN2 | ENSG00000065243.18 | 0.62 |
| MAP3K2 | ENSG00000169967.16 | 0.62 |
| NSD1 | ENSG00000165671.18 | 0.62 |
| BCLAF1 | ENSG00000029363.15 | 0.62 |
| RBM12 | ENSG00000244462.7 | 0.62 |
| THRAP3 | ENSG00000054118.13 | 0.61 |
| EP300 | ENSG00000100393.9 | 0.61 |
| ZMYM4 | ENSG00000146463.11 | 0.61 |
| ZNF562 | ENSG00000171466.9 | 0.61 |
| WASF2 | ENSG00000158195.10 | 0.61 |
| WAC | ENSG00000095787.21 | 0.61 |
| USP37 | ENSG00000135913.10 | 0.61 |
| DDX46 | ENSG00000145833.15 | 0.61 |
| C16orf72 | ENSG00000182831.11 | 0.6 |
| HIPK1 | ENSG00000163349.21 | 0.6 |
| CACUL1 | ENSG00000151893.14 | 0.6 |
| UBN1 | ENSG00000118900.14 | 0.6 |
| TRAPPC10 | ENSG00000160218.12 | 0.6 |
| USP24 | ENSG00000162402.12 | 0.6 |
| OTUD4 | ENSG00000164164.15 | 0.59 |
| LTN1 | ENSG00000198862.13 | 0.59 |
| LATS1 | ENSG00000131023.12 | 0.59 |
| MIER1 | ENSG00000198160.14 | 0.59 |
| DICER1 | ENSG00000100697.14 | 0.59 |
| UTP11L | ENSG00000183520.11 | 0.59 |
| HP1BP3 | ENSG00000127483.17 | 0.59 |
| FBXW2 | ENSG00000119402.16 | 0.59 |
| GPATCH8 | ENSG00000186566.11 | 0.59 |
| RLIM | ENSG00000131263.12 | 0.59 |
| MORC3 | ENSG00000159256.12 | 0.59 |
| AP1G1 | ENSG00000166747.12 | 0.58 |
| SP1 | ENSG00000185591.9 | 0.58 |
| SRCAP | ENSG00000080603.16 | 0.58 |
| CREBBP | ENSG00000005339.12 | 0.58 |
| ZNF592 | ENSG00000166716.9 | 0.58 |
| EFCAB14 | ENSG00000159658.10 | 0.58 |
| KDM5A | ENSG00000073614.11 | 0.58 |
| RC3H2 | ENSG00000056586.15 | 0.58 |
| CCNT1 | ENSG00000129315.9 | 0.58 |
| 7-Mar | ENSG00000136536.14 | 0.58 |
| KDM4A | ENSG00000066135.12 | 0.58 |
| RNF111 | ENSG00000157450.15 | 0.58 |
| ZNF805 | ENSG00000204524.6 | 0.58 |
| CTDSPL2 | ENSG00000137770.13 | 0.58 |
| ARID1A | ENSG00000117713.17 | 0.58 |
| ZNF148 | ENSG00000163848.18 | 0.58 |
| TRIP11 | ENSG00000100815.12 | 0.58 |
| ANKRD17 | ENSG00000132466.17 | 0.58 |
| TET3 | ENSG00000187605.15 | 0.58 |
| SCYL2 | ENSG00000136021.17 | 0.58 |
| ZFP91 | ENSG00000186660.14 | 0.58 |
| NAA15 | ENSG00000164134.12 | 0.58 |
| ASH1L | ENSG00000116539.10 | 0.58 |
| PRDM10 | ENSG00000170325.14 | 0.58 |
| DYRK1A | ENSG00000157540.19 | 0.58 |
| CEP350 | ENSG00000135837.15 | 0.58 |
| MCM9 | ENSG00000111877.17 | 0.58 |
| BAZ2A | ENSG00000076108.11 | 0.58 |
| ZSCAN29 | ENSG00000140265.12 | 0.57 |
| TNKS2 | ENSG00000107854.5 | 0.57 |
| PDCD6IP | ENSG00000170248.13 | 0.57 |
| TAF1 | ENSG00000147133.15 | 0.57 |
| SYNCRIP | ENSG00000135316.17 | 0.57 |
| SBNO1 | ENSG00000139697.11 | 0.57 |
| ZSCAN20 | ENSG00000121903.14 | 0.57 |
| RBM12B | ENSG00000183808.11 | 0.57 |
| DDX6 | ENSG00000110367.11 | 0.57 |
| RP11-447D11.3 | ENSG00000280173.1 | 0.57 |
| AP4E1 | ENSG00000081014.10 | 0.57 |
| WNK1 | ENSG00000060237.16 | 0.57 |
| ANKRD13C | ENSG00000118454.12 | 0.57 |
| DENND6A | ENSG00000174839.12 | 0.57 |
| IREB2 | ENSG00000136381.12 | 0.57 |
| PDS5A | ENSG00000121892.14 | 0.57 |
| SCAF11 | ENSG00000139218.17 | 0.57 |
| HIF1AN | ENSG00000166135.13 | 0.57 |
| TMOD3 | ENSG00000138594.12 | 0.57 |
| RP11-159G9.5 | ENSG00000229729.6 | 0.57 |
| RBM27 | ENSG00000091009.7 | 0.57 |
| NUP50 | ENSG00000093000.18 | 0.57 |
| SP3 | ENSG00000172845.13 | 0.57 |
| UBE4A | ENSG00000110344.9 | 0.57 |
